# NMR-Based Quantification of Collagen Content in Protein Hydrolysates

**DOI:** 10.1021/acs.jafc.5c13447

**Published:** 2026-02-09

**Authors:** Greta Nardini, Kristian Hovde Liland, Guido F. Pauli, Sileshi Gizachew Wubshet, Alistair L. Wilkins, Frode Rise, Matthias Niemitz, Nils Kristian Afseth, Kenneth Aase Kristoffersen

**Affiliations:** † Faculty of Chemistry, Biotechnology and Food Science, 56625 Norwegian University of Life Sciences, P.O. Box 5003, NO-1432 Ås, Norway; ‡ Faculty of Science and Technology, Norwegian University of Life Science, P.O. Box 5003, NO-1433 Ås, Norway; § Pharmacognosy Institute & Department of Pharmaceutical Sciences, Retzky College of Pharmacy, 14681 University of Illinois Chicago, Chicago, Illinois 60612, United States of America; ∥ Fisheries and Aquaculture Research, 548481 Nofima—Norwegian Institute of Food, P.O. Box 210, NO-1431 Ås, Norway; ⊥ Chemistry Department, 3717 The University of Waikato, 3240 Hamilton, New Zealand; # Department of Chemistry, University of Oslo, P.O. Box 1033, Blindern, NO-0315 Oslo, Norway; ¶ NMR Solutions Ltd., 70110 Kuopio, Finland

**Keywords:** food protein hydrolysates, hydroxyproline, collagen quantification, qHNMR spectroscopy, ^1^H-functionalized spin analysis (HifSA), QM-qHNMR

## Abstract

Certain functional food and dietary supplement ingredients contain bioactive peptides whose bioactivity is determined by their chain length, composition, and specific sequence of the amino acids. This study investigated collagenous peptides from the valorization of poultry side streams, which, for example, have inhibitory activities against therapeutic targets for the management of blood pressure and blood glucose. To overcome the specificity challenges of conventional amino acid analysis, the aim was to establish and validate a quantitative ^1^H NMR (qHNMR) method for the quantification of hydroxyproline as a marker of collagen content in poultry hydrolysates. Indeed, qHNMR provides a rapid structure-specific quantification, and it is highly informative, reproducible, and accurate. The effect of different acidic solvents was investigated using quantum mechanics (QM)-based ^1^H iterative functionalized spin analysis (HifSA). Comparison of conventional integration (int-) with QM-qHNMR revealed limitations of the former approach and highlighted the increased accuracy and specificity of QM-qHNMR.

## Graphical abstract

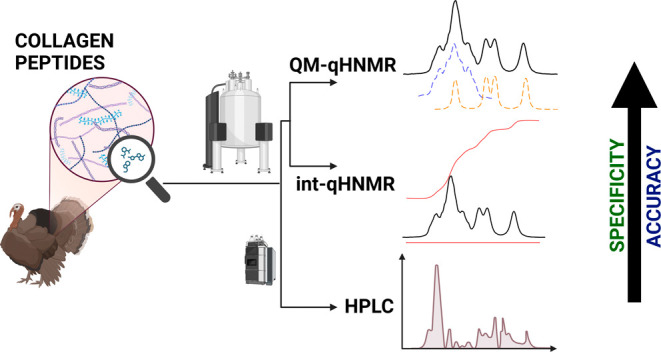

## 1 Introduction

Protein hydrolysates have been gaining popularity due to their potential health-promoting properties and role in a sustainable circular economy, and due to their potential to maximize the utility of food production side-stream lines.^1–3^ Protein hydrolysates are often produced via enzymatic protein hydrolysis (EPH) of protein-rich biomasses, and the hydrolysates are generally complex mixtures of thousands of peptides of different sizes and amino acid sequences. Relevant biomasses for EPH include side streams from various food industries, such as the agriculture, fish, meat, and dairy sectors. The protein hydrolysates contain peptides with potentially diverse purported bioactivities such as immune-enhancing, anti-hypertensive, anti-oxidative, anti-diabetic, anti-obesity, and hypocholesterolemic effects.^1^ The hydrolysates can also play important roles as functional food or dietary supplement ingredients when separated and isolated as pure products. Considering global meat production, poultry is a promising source of edible side streams because it is one of the most consumed meats in the world and one of the most sustainable on a CO_2_-eq per kg protein basis.^4^ Poultry carcasses can be used to produce protein-rich hydrolysates. Poultry hydrolysates have demonstrated, for example, angiotensin-1-converting enzyme (ACE-1) and dipeptidyl-peptidase 4 (DPP4) inhibitory activities, two therapeutic targets for the management of blood pressure and blood glucose.^5^

Poultry protein hydrolysates contain a significant portion of collagen.^4^ Collagens have characteristic triple helix structures. Each α-chain of the triple helix contains the repetitive unit glycine (Gly)-X-Y, where X and Y are mostly occupied by proline (Pro) and hydroxyproline (Hyp), respectively.^6,7^ Since Hyp is a modified amino acid, almost exclusive to collagen proteins, it is considered a suitable biomarker for the determination of collagen content.^8,9^ The hydroxylation level of Pro is known to be different in collagens of different origins. The melting temperature of the collagen triple helix, and thus the stability of the collagen structure, is directly proportional to the Hyp content.^10,11^ For example, for poultry, the collagen content is calculated based on the assumption that collagen proteins contain 13.5% Hyp (w/w %), as established for warm-blooded animals.^9,12^ In cold-blooded animals, such as cod, the collagen is reported to contain lower levels of Hyp, approximately 6.8%.^13^

The amino acid composition, and especially the Hyp content, is an essential attribute of poultry hydrolysates as the Hyp content dictates a range of parameters, from potential bioactivities to functional properties. According to EU Commission (EC) Regulation No. 152/2009, the total amino acid composition is determined through 24 h of acidic hydrolysis of the material, followed by neutralization, separation by ion exchange chromatography, reaction with ninhydrin, and photometric detection. As these analyses are laborious and time-consuming, they are typically performed by accredited laboratories for cost-effectiveness.^14,15^ Notably, quantification requires a calibration standard for each analyte. Accordingly, errors vary between amino acids and are typically about 20% for Hyp, as reported by commercial analytical laboratories.

A faster option is a colorimetric method specifically developed for Hyp.^16^ This assay can be performed within 5 h but requires expensive reagents and yields uncertainties between 10 and 50% depending on the operator and material background signal.^7^

Spectroscopic alternatives for collagen analysis have recently been investigated. One study used dry film Fourier transform infrared (FTIR) spectroscopy and a multivariate calibration model, specifically hierarchical cluster-based partial least-squares (HC-PLS) regression, to quantify collagen based on vibrations in the amide bands caused by Pro and Hyp. The distinct FTIR spectral patterns allowed relative quantification based primarily on the tertiary amide vibrations of Pro and Hyp when these residues were incorporated into a polypeptide chain.^12^ This approach provided acceptable results; however, the chemical resolution of FTIR spectroscopy is limited as free amino acids are not detected. On the contrary, nuclear magnetic resonance (NMR) provides structural information at the atomic level; it is inherently quantitative (qNMR) and can perform multitarget analysis.^17–19^ In fact, another study used qNMR after total hydrolysis and without further sample preparation: within a single analysis, the authors quantified Hyp, Gly, and alanine (Ala).^20^ One downside of the method was the need for high concentrations of deuterated acid at 6 M, which served as a shifting agent to reduce the peak overlap and enable quantitation. As expected, qNMR had major advantages over the other methods. It exhibited high reproducibility, specificity, and accuracy, which were in line with quantitative ^1^H NMR (qHNMR) being rated as a metrological method.^21^

Nevertheless, several challenges remain to be addressed when establishing highly precise qHNMR methods for poultry hydrolysate analysis. First, the physicochemical nature of the hydrolysates is captured by NMR but deviates from classical recommendations for qHNMR conditions because of variable salt content that requires careful adjustment in the instrument (i.e., probe tuning and matching). Furthermore, peak overlap remains a major source of uncertainty due to the typically highly crowded ^1^H NMR spectra, which limits the specificity achievable by integration (int-qHNMR). Finally, high-molecular-weight (MW) molecules give rise to baseline imperfections. These high-MW molecules result from incomplete hydrolysis and due to their short *T*_2_ relaxation time constants give broad resonances.^22–24^

To address both the specificity and accuracy challenges in the qHNMR analysis of EPHs, this study utilized a quantum mechanics (QM)-based evaluation of qHNMR spectra. This approach employs ^1^H iterative functionalized spin analysis (HifSA), a computational QM-based spin analysis (QMSA), for a detailed analysis of ^1^H-qNMR spectra.^25,26^ This analysis utilizes HifSA profiles of the target amino acid as ID and quantitation references.

HifSA involves the QM foundation and, through computation, yields the true spin parameters (chemical shifts (δ), coupling constants (*J*), and line widths (υ [syn. ω_1/2_])) of a given molecule. This concept was established in 2013 through an evolution of the classical concept of full spin analysis (FSA), which was introduced as early as the discovery of the NMR effect.^27^ HifSA captures both the qualitative and quantitative properties of ^1^H NMR spectra simultaneously. Importantly, HifSA resolves peak overlap and fully considers all higher-order effects, both of which continue to challenge the interpretation even of ultrahigh-field NMR spectra. The known utility of ^1^H NMR spectra in metabolomic analysis bodes well for their broader implementation in the analysis of complex mixtures, such as EPHs.^28–32^ Also, from an economic perspective, qNMR analysis continues to gain acceptance as an alternative method supported by high-throughput instrumentation and the increasing capabilities of cryogen-free benchtop instrumentation.^19,25^

The present study investigated the effect of acid on the ^1^H NMR spectra and compared the quantitative outcomes obtained via classical integration with those of QM-assisted quantification. The study goal was not only to overcome the limitation of classical int-qHNMR but also to reduce sample preparation requirements and simplify calibration compared with other methods such as FTIR spectroscopy. The new qHNMR method for collagen in poultry hydrolysates was also validated. Collectively, the study aimed at establishing an easy-to-use and rapid qHNMR method, with increased specificity and accuracy over the previous approaches, to set the stage for the broader implementation of EPH amino acid analysis by qHNMR.

## 2 Materials and Methods

### 2.1 Raw Materials and Chemicals

Hydroxyproline (Hyp; *trans*-4-hydroxy-l-proline, C_5_H_9_NO_3_); the certified reference material (CRM) dimethyl sulfone (DMSO_2_; 99.96%, (CH_3_)_2_SO_2_) used as the internal calibrant (IC); and the deuterated solvents, deuterium chloride 35% (DCl) and deuterium oxide with 1% 3-(trimethylsilyl)-1-propanesulfonic acid (D_2_O with designated 1% DSS and quantitatively determined to be 0.90%; 99.9 atom % D), used as an internal standard for chemical shift (0.000 ppm), were purchased from Sigma-Aldrich (St. Louis, MO, USA). The amino acid glycine (Gly, C_2_H_5_NO_2_) was obtained from AppliChem GmbH (Darmstadt, Germany), while D_2_O (99.8 atom % D) was from Thermo Fisher Scientific (Waltham, MA, USA). HCl 37% for hydrolysis was purchased from VWR (Radnor, PA, USA).

Turkey hydrolysate samples were selected from previously published studies.^4,7^ Two samples containing lower collagen amounts served as blanks (TC_A, TC_B), while three hydrolysates containing medium to high collagen amounts served as analyte samples (TC_C, TC_D, and TC_E). Table 1 presents an overview of the samples used in this study; additional information can be found in the Supporting Information Table S1.

**Table 1:** Hyp content (w/w %) and corresponding collagen conversion (w/w %) as well as ash (w/w %) of the turkey hydrolysate samples used in this study (See Table S1 for additional information).

Sample name	Hyp (%)	Collagen^a^ (%)	Ash (%)
TC_A	0.0^b^	0.3	N.D.^d^
TC_B	0.1^b^	0.7	N.D.^d^
TC_C	2.35^c^	17.4	N.D.^d^
TC_D	4.63^c^	34.3	5.01^c^
TC_E	4.82^c^	35.7	4.69^c^

a Calculated with the conversion factor 7.4 for warm-blooded animals (collagen contains 13.5% (w/w %) of Hyp).^12^

b Determined by the colorimetric method.^7^

c Determined by Eurofins Scientific according to ISO 13903:2005, EU 152/2009 (F) for Hyp, and the NMKL 173 method for ash.^4^

d N.D.: not determined.

### 2.2 Sample Preparation

All volumetric and gravimetric work was performed at room temperature. Three different types of samples were prepared: one set of Hyp solutions with known concentrations from 0.00 to 6.0 M DCl for the solvent study; one set with three hydrolysate samples; one set for method validation containing only Hyp and Gly as reference analyte; a blank sample with the hydrolysate background; and several different analyte samples with known amounts of Hyp from previous studies or added to the samples.

#### 2.2.1 Solvent Study

For the solvent study, seven Hyp solutions with different ionic strengths were prepared as follows. First, a 28.6 mM Hyp stock solution was prepared by weighing 10.36 mg of Hyp on a microbalance (CP2P, Sartorius) and dissolving it in 2.551 mL of D_2_O, with addition of 0.214 mL of D_2_O with 1% DSS. Next, 273 μL of this Hyp stock solution were transferred into each 5 mm NMR tube (Wilmad Bruker SampleJet), and different proportions of D_2_O and DCl liquids were added to obtain a total volume of 600 μL to create the seven acidic solutions: 0.00, 0.50, 0.61, 0.99, 1.5, 3.0, 6.0 M DCl, respectively. All the samples had a final theoretical concentration of 13.0 mM Hyp and 1.62 mM DSS. Biological duplicate samples for each acidic solution were created.

#### 2.2.2 Hydrolysate Samples

Hydrolysate samples were analyzed to explore differences in quantification between qHNMR and HPLC. Turkey hydrolysates produced using food-grade commercial protease preparations, i.e., Flavorzyme, Corolase, and Alcalase (TC_C, TC_D, and TC_E, respectively), were used to span the variation of collagen content in the hydrolysates. The freeze-dried powder of the enzymatically hydrolyzed poultry material was dissolved in 1.0 M HCl to produce an exact 50.0 mg/mL solution. Acid was used instead of water to avoid gel formation. Next, the samples were further hydrolyzed in an aluminum heating block (VWR) with 6.0 M HCl for 24 h at 110 °C, as stated by the ANNEX III para. F-G of EU Commission (EC) regulation no. 125/2009. Subsequently, the samples were reduced to dryness using nitrogen gas flow over 4 h at 60–40 °C, reconstituted in 600 μL of 0.61 M DCl with 1.62 mM DSS, and filtered directly into the NMR tubes with 0.45 μm water-wettable polytetrafluoroethylene filters (wwPTFE, Acrodisc One, Pall Corporation). The tubes were closed with caps, which were sealed with parafilm. The three turkey hydrolysates were prepared and analyzed once.

#### 2.2.3 Method Validation

The validation of the method included different assessments: recovery, linearity with determination of limit of quantification (LOQ) and detection (LOD), precision, stability, and repeatability.

The recovery was tested using the hydrolysate sample TC_A containing low collagen (<1 w/w %) as a blank, and by spiking low, medium, and high levels of Hyp and Gly, in triplicate: 50.0% (0.236 mg of Hyp and 0.375 mg of Gly), 200% (0.944 mg of Hyp and 1.50 mg of Gly), and 500% (2.36 mg of Hyp and 3.75 mg of Gly). Prior to spiking, the samples were hydrolyzed with 6.0 M HCl for 24 h at 110 °C as described above. After spiking, the samples were reduced to dryness, reconstituted in the deuterated solvent, and filtered into the NMR tubes as for the other hydrolysate samples. A reference sample containing only 13.0 mM Hyp and 24.4 mM Gly was also prepared to assess the differences due to the sample matrix effect of the hydrolysates.

Linearity was assessed using the hydrolysate sample TC_B, which contained <1 w/w % collagen and, thereby, low Hyp. Duplicate samples were spiked with 6 different amounts of Hyp to cover the typical concentration range of poultry hydrolysates: 2.50, 5.00, 7.50, 10.0, 12.5, and 15.0 mM.^7^ Simultaneously, the same amounts of Gly were spiked to yield a parallel assessment. The samples were hydrolyzed and prepared as described above.

The LOD and LOQ were determined following eq 1 according to ISO 11843–2:2000 and eq 2 according to EU Commission (EC) regulation 333/2007, respectively.

1 $$x_{LOD}=3.8\cdot \frac{s_{y,x}}{b}\cdot \sqrt{1.1+\frac{{\mathrm{x̅}}^{2}}{\sum_{i=1}^{n}{\left(x_{i}-\mathrm{x̅}\right)}^{2}}}$$

2 $$x_{LOQ}=3.3\cdot x_{LOD}$$

where *x*_LOD_ is the limit of detection, *x*_LOQ_ is the limit of quantification, *s*_*y*,*x*_ is the standard deviation of the residuals, *b* is the slope of the calibration curve, *x̅* is the mean calibration level, and *x*_*i*_ is the content value of the analyte at the calibration level *i*. The values were furthermore compared with the corresponding signal-to-noise (S/N) values calculated by the au program *sinocal* in TopSpin. The au program used the best 2 ppm noise region available.

Precision, stability, and repeatability were estimated using the hydrolysate sample TC_E. The material was hydrolyzed with acid, dried, resolubilized, and filtered as described above. The precision was estimated in terms of sample preparation (precision intersample) with 6 samples prepared independently. Six replicate measurements of the same sample (precision instrument) as well as six independent processing runs of the same raw qHNMR data (precision processing) were performed. Stability was evaluated by measuring the same sample over time, starting from its preparation, at 30 min, 2.0 h, 3.0 h, 5.5 h, 10.5 h, 20 h, 40 h, 3 days, 1 week, 2 weeks, 3 weeks, and 9 weeks (2 months). Repeatability, representing interday variability, was evaluated by preparing and analyzing two independent samples three times, one pair per week.

The above-mentioned validation parameters were evaluated as relative standard deviation (RSD; eq 3), calculated as the percentage ratio of the standard deviation (σ) to the sample mean (*x̅*), and as relative error (error %; eq 4), calculated as the percentage ratio of the absolute error to the expected concentration.

3 $$RSD=100\cdot \frac{\sigma }{\mathrm{x̅}}$$

4 $$error\;\%=100\cdot \frac{\left|spiking\;conc-\left(conc\;spiked\;sample-conc\;unspiked\;sample\right)\right|}{spiking\;concentration}$$

### 2.3 NMR Instrumentation

NMR measurements were performed on a Bruker NMR spectrometer consisting of a 400 MHz magnet (Ascend; 400 MHz for ^1^H) with an Avance III HD console and equipped with a 5 mm broad-band probe (BBO) maintained at 25 °C (298 K) (Bruker BioSpin AG, Fällanden, Switzerland). The instrument was operated with TopSpin v.3.6.5 software (Bruker BioSpin GmbH, Ettlingen, Germany).

### 2.4 Inversion–Recovery Experiments (*T*_1_)

The *T*_1_ spin–lattice relaxation time constants were determined for all hydrogens using an inversion–recovery pulse sequence (180°−τ–90°). The τ interval was set to 16 data points between 0.01 and 40 s, with the number of scans (NS) set to 8 and dummy scans (DS) to 4 for each interval. The 90° pulse (*P*_1_) was optimized by Topspin’s automatic pulse calibrating program *pulsecal*.^33^ The relaxation delay (*D*_1_) was set to 40 s, and 16384 data points were acquired at a spectral width (SWH) of 8013 Hz. The FID was processed with a 0.3 Hz line broadening (LB) and an exponential window multiplication (EM) function prior to Fourier Transformation (FT). The *T*_1_ values were calculated using the processed spectra via the relaxation analysis module of the Bruker Topspin software.

### 2.5 qHNMR Experiments

The instrument was locked via the deuterium channel, and a 5 min waiting time was used before shimming to equilibrate the temperature of the sample in the magnet.^34^ The shimming procedure, according to the Automatic Shimming User Manual v. 008 (March 30, 2023 Bruker BioSpin GmbH), employed both the classical TopShim command and the TopShim command optimized for convection effects (see Supporting Information Section S2) until the full width at half-maximum (FWHM) and the line shape quality factor (LQF) were considered acceptable with values ≤0.3 and ≤0.450, respectively. Considering the salt content of the samples, to avoid stressing the piezoelectric motors that control the tuning and matching (T/M) capacitors, T/M were adjusted manually to approximate the automatic “exact” setting (the proton frequency, 400 MHz, and 0 [rel.] intensity) as closely as possible.^35,36^

The qHNMR spectra were recorded with a standard single-pulse sequence using a 90° pulse optimized by *pulsecal*.^33^ The acquisition parameters were 16 NS, 4 or 0 DS, 65,536 data points, 8013 Hz SWH, and 4.09 s acquisition time (AQ); digitization mode was digital or baseopt. The interpulse delay *D*_1_ was set to maintain a pulse repetition time (Tr) [AQ + *D*_1_] as close as possible to at least 5 × *T*_1_ for all signals of interest.

After acquisition, the spectra were processed using both TopSpin v.4.3.0 and MestReNova v.15.1.0 (MNova; Mestrelab Research, Santiago de Compostela, Spain).

In TopSpin, the spectra were processed with zero filling (2×) to 131,072 data points, followed by EM with 0.3 Hz LB and FT. Automatic phase and baseline corrections were applied, and the spectra were manually referenced to DSS (0.000 ppm). All the spectra were integrated in series for all of the peaks of interest, and bias and slope were corrected manually. According to Griffiths and Irving, an integral that extends to at least 24 times the peak half-width in both directions encloses 99% of the total area for a Lorentzian peak.^37^ This integral range could not be used because the widest range applicable to all of the peak patterns was ±10 Hz (0.03 ppm); therefore, this range was used in the present work.

In MNova, the spectra were subjected to apodization with exponential (EM) and Gaussian (GB) window functions set to −0.3 and 0.3 Hz (mild Lorentzian–Gaussian enhancement), respectively; zero filling (2×) to 131,072 data points with forward linear prediction, and multipoint baseline correction.^26,38,39^ The NMR spectra were exported as .jdx (JCAMP-DX) files for further analysis in Cosmic Truth (CT; NMR Solutions Ltd., Kuopio, Finland). HifSA profiles were generated as described by Achanta et al., serving both qualitative and quantitative purposes.^25^

### 2.6 Quantification

Two quantification methods were compared: the classical integration method using TopSpin software and the QM-based method using spectra calculated by CT (QMSA approach). In the QM-based method, the quantitative measures are derived from the populations of the spin systems fitted by iterative spectral analysis, HifSA, in CT.^26,27,40^

All processed qHNMR spectra were analyzed with the ERETIC2 module of TopSpin v.4.3.0. The concentrations were calculated automatically according to eq 5 from the TopSpin ERETIC2 User manual v. 001 (June 29, 2016, Bruker Corporation).^41^

5 $$C_{u}=k\cdot C_{R}\frac{A_{u}\cdot n_{R}\cdot T_{u}\cdot \theta_{u}}{n_{u}\cdot A_{R}\cdot T_{R}\cdot \theta_{R}}$$

where the indices “*u*” and “*R*” stand for unknown sample and reference, respectively, *C* for the concentration, *A* for the integral value, *n* for the number of scans used for the experiments, *T* for the temperature, θ for the pulse length, and *k* for a correction factor considering the use of different receiver gain (RG) values or incomplete relaxation. A reference sample was accurately prepared using the microbalance and electronic autopipettes (Picus, Sartorius). The exact amount of DMSO_2_ was used for external calibration (EC). It contained 2.01 mM DMSO_2_ and 1.67 mM DSS in 0.600 mL of D_2_O specifically.

A subset of spectra was also processed with MNova and analyzed by using CT software. The populations of the generated HiFSA profiles were then used for the quantification, entering the values in the publicly available ECIC calculation spreadsheet (see Supporting Information Section S3).^42^

## 3 Results and Discussion

We studied turkey hydrolysates due to their potential as functional food ingredients, particularly in relation to their collagen content.^7^ Amino acid composition and length of the peptides are essential for their functional activities, and the amount of collagen peptides was determined based on Hyp content (Figure 1). A qHNMR method for collagen quantification is desirable due to its potential development into a fast and easy-to-use tool for peptide characterization.^20^ Thus, in the present study, the effect of a range of salt concentrations on the sample properties and their influence on the quantification was analyzed. After the method was validated with an optimized salt concentration, different approaches for quantification were explored in more detail.

**Figure 1:**
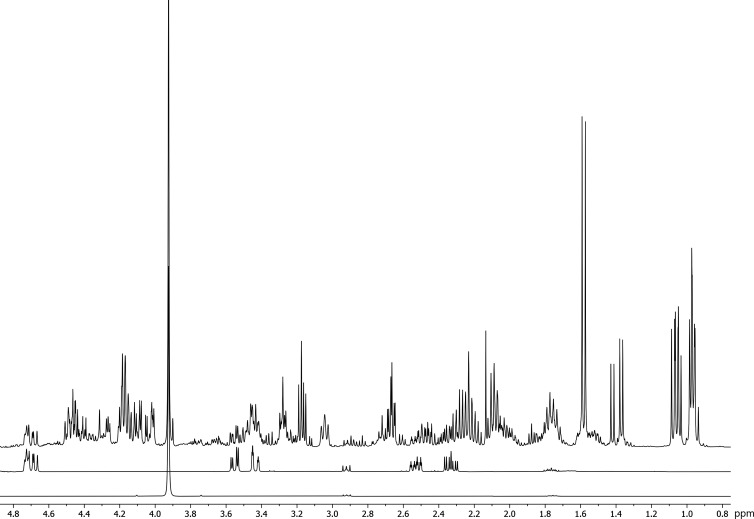
Turkey hydrolysate (top), hydroxyproline (Hyp; middle), and glycine (Gly; bottom) ^1^H NMR spectra (400 MHz, 0.8–4.8 ppm region). The turkey hydrolysate was totally hydrolyzed in acid. The collagen content in the hydrolysate can be determined based on Hyp content, and Gly is another essential amino acid of the collagen unit. All spectra were recorded in 0.61 M DCl and referenced to 0.000 ppm using DSS.

### 3.1 Effects of Different Solvent Acidity

Unlike pure analyte solutions, protein hydrolysates require complete acidic hydrolysis to release the constituent amino acids (i.e., Hyp) from peptides and protein fragments. Therefore, understanding the effect of different acidic conditions on the NMR spectra and hence the quantification of Hyp is essential. The effect of the different ionic strengths on the qHNMR spectra was assessed using solutions containing 13.0 mM Hyp (Figure 2). These solutions mimicked the EPH samples for studying the consequences of acid concentration on the qHNMR spectra, the NMR-relevant physiochemical properties of the solutions, instrument behavior, and the impact on quantitation.

**Figure 2:**
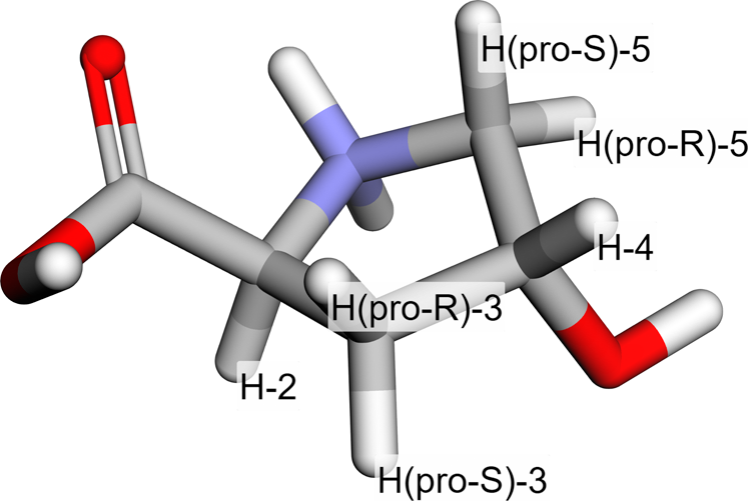
3D structure of the amino acid hydroxyproline (Hyp), with numbered protons. The relative geometries of the protons are important for the H, H spin-coupling system of the molecule.

**Table 2:** Observed chemical shift (δ) range of the hydroxyproline (Hyp) protons and the residual water peak (HDO), presented as averages of the different acidic solutions, varying between 0.00 and 6.0 M DCl

	Chemical shift (δ)	
Atom name	Average (ppm)	Range (ppm)
HDO	5.483	2.350
H-4	4.721	0.110
H-2	4.649	0.422
H(*pro-S*)-5	3.549	0.142
H(*pro-R*)-5	3.436	0.154
H(*pro-S*)-3	2.517	0.146
H(*pro-R*)-3	2.313	0.227

The shift values are influenced by the various acid concentrations because, due to the p*K*_a_ of the molecules in solution, the protonation state of the pH-sensitive functional groups affects the shielding and deshielding status of all neighboring atoms.^43^ A uniform deshielding effect was observed for all of the resonances in the spectrum (Supporting Information Figure S1), as all peak patterns shifted downfield. Table 2 shows that the chemical shift changes cannot be considered negligible for any resonance; while it is only 0.1–0.2 ppm for most protons, it is 0.4 ppm for proton H-2. The proton H-2 shifts from 4.335 ppm in 0.00 M DCl to 4.758 ppm in 6.0 M DCl due to the protonation of the amino and carboxyl groups. The values for all of the chemical shifts are presented in Supporting Information Table S2. It is worth noting that the most affected peak in the spectrum is the residual HDO peak, which shifts 2.350 ppm in total. In fact, the distance between the HDO and the DSS reference peak is a direct measure of the pH. The concentration of ions in the solution linearly correlates with the change in their chemical shifts (*R* ^2^ = 0.995, Supporting Information Figure S2). This can be explained by the decrease in the magnetic shielding effect due to the decrease in the ratio of electrons per ^1^H nucleus in the generation of hydronium ions.^44^

Importantly, while the chemical shifts change with the acid concentration, the coupling constants (*J*) remain unaffected. The exact *J* values obtained by HifSA vary only between 0.02 and 0.30 Hz (Supporting Information Table S3). Considering that this variation is well below the average line width of the spectra, it becomes negligible. Collectively, this proves the constant nature of the *J* couplings and their high consistency across different solvent ionic strengths. As their name implies, *J* coupling constants are truly constant and therefore can be used for identifying a molecule unambiguously. This holds true provided that changes in the experimental conditions do not alter the molecule’s conformational space to an extent that affects the *J* couplings. In contrast, the absolute chemical shifts (δ) require close to identical NMR conditions to be meaningful for this purpose.^25^

The properties of the Hyp solutions are also influenced by the solvent used, as shown in Figure 3. Specifically, protons exhibit a shorter *T*_1_ relaxation time as the acid concentration (hydronium ions) augments, which increases the formation of intermolecular hydrogen bonds.^45^ Conversely, part of the radiation is dissipated in highly acidic samples, increasing the P_1_ pulse duration required to obtain a 90° flip angle. In a scenario where a shorter *T*_1_ would allow the use of an 18% shorter *D*_1_ value for samples in 6.0 M acid (4.9 s for D_2_O and 4.0 s for 6.0 M DCl), the length of *P*_1_ would quadruple under these conditions (9.7 μs for D_2_O and 29.1 μs for 6.0 M DCl). Considering the magnitude difference of these two variations, the total time for each cycle (*P*_1_ + AQ + *D*_1_) could be reduced at higher acid concentrations due to the shorter *T*_1_.

**Figure 3:**
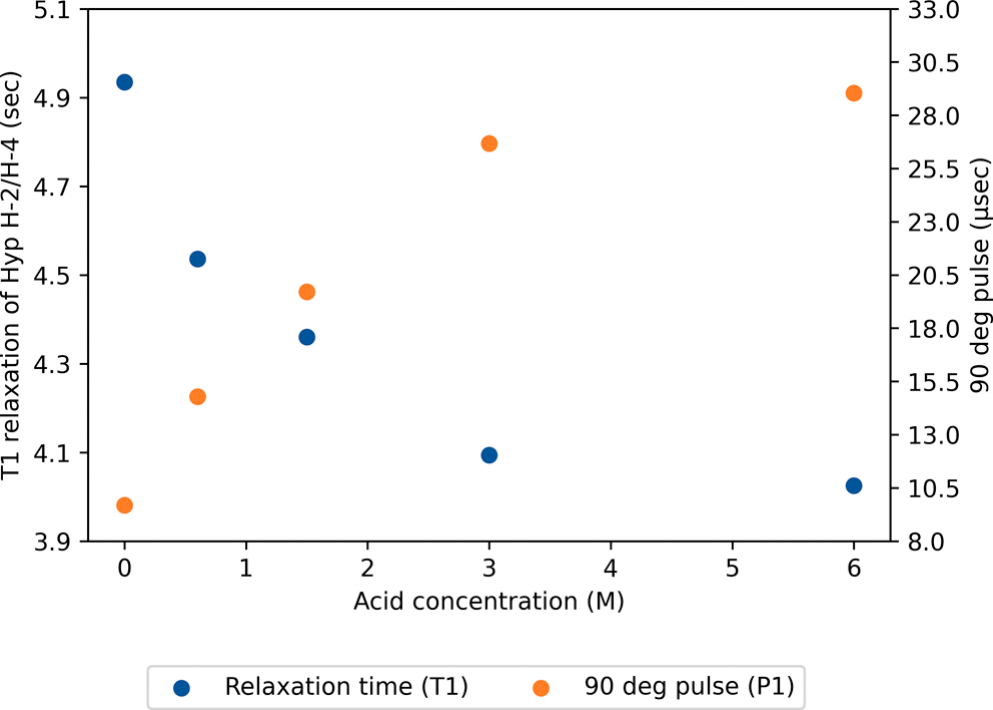
Relaxation times *T*_1_ (blue) and 90 deg pulse length *P*_1_ (orange) of the atoms H-4 and H-2 in hydroxyproline (Hyp) at the acid concentrations tested (0.00–6.0 M DCl). Both *y* axes are shifted from the axis origin (0, 0); 3.9 s for the *T*_1_ axis and 8.0 μs for the *P*_1_ axis. The relaxation time axis is scattered and 6 orders of magnitude greater compared to the 90 deg pulse axis for graphical purposes; they represent a total of 1.2 s and 25 μs, respectively.

Furthermore, the presence of acid and, therefore, ions in solution causes an increase in the surface tension, which in turn leads to lower evaporation rates. While this does not affect sealed tubes directly, it is useful when concentration determinations are performed as the composition of the sample is more stable.^46–48^ On one side, ionic concentration prevents evaporation. On the other side, the ions in a solution contribute to line broadening. This also hampers tuning and matching of the probe (Supporting Information Figure S3 and Table S4), giving rise to unwanted deviations in signal intensity.

To summarize the ionic strength effects up to this point, it can be concluded that, although acid can be used to shorten *T*_1_ and decrease the total time of acquisition and it can be used as a shifting agent for moving peaks of interest within the spectrum and minimizing peak overlap, the total ionic strength of the solution highly affects the NMR experiment and its quantitative nature. This is relevant for food and EPH samples, as studied here, since processing animal side-streams often results in relatively high ash content derived from bones, which should be carefully considered. As the ash content exhibits high variability, this can influence the quantitative analysis and product profiling of these materials by qHNMR.^49^ For these reasons, the aspect of the ionic strength was investigated further.

Figure 4 shows the effects of the acid content on the qHNMR analysis. Samples containing 13.0 mM (12.9 mM when considering the declared purity) Hyp and 1.62 mM DSS were prepared in duplicate (*n* = 2) for all tested acidic solutions (0.00–6.0 M DCl). For each sample, three independent ^1^H NMR spectra were acquired and processed to enable statistical analysis (*n* = 6). Quantification used the classical integration method with external calibration (EC), calculated by the ERETIC2 method for all the Hs of Hyp and for DSS. The results highlight an overestimation of Hyp content, within 3σ, when increasing the acid concentration and an underestimation, within 1σ, when using only D_2_O. Overall, the comparison of the seven acidic solutions shows an increasing trend from 0.00 to 6.0 M DCl for all the peak patterns used to determine the concentration of the analyte. This suggests that the accuracy of the quantification by integration decreases as ion concentration increases.

**Figure 4:**
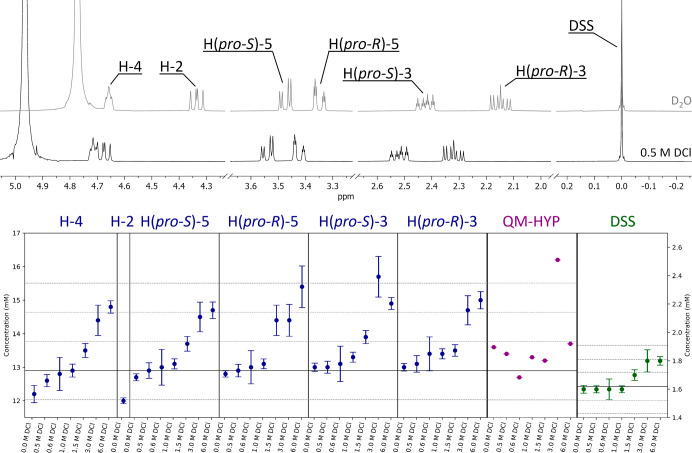
Spectrum of hydroxyproline (Hyp) in D_2_O with added DSS (residual HDO peak at 4.772 ppm; above, gray) and 0.50 M DCl (residual HDO at 4.965 ppm; below, black). The scatter plot shows the concentration calculated with ERETIC2 for all investigated acid concentrations (0.00–6.0 M DCl), with each box representing the quantification of Hyp based on the corresponding H atom (blue; *n* = 2^a^, *n* = 6^b^), from left to right: H-4, H-2, H­(*pro-S*)-5, H­(*pro-R*)-5, H­(*pro-S*)-3, H­(*pro-R*)-3. The second box from the right shows the quantification of Hyp via HifSA profiles calculated according to QM principles, using the population with ECIC (purple; *n* = 1^b^). The rightmost box shows the quantification of DSS, the internal calibrant (IC), via the ERETIC2 method (green; *n* = 2^a^, *n* = 6^b^). The theoretical concentrations (black solid lines) of 12.9 mM for Hyp and 1.62 mM for DSS and the 1–2–3 σ (gray dashed lines) are represented to highlight the statistical relevance of the concentration variation. (a) Biological replicates. (b) Number of spectra used for the quantification.

It is important to consider that only the peak patterns of H-4 in D_2_O and of H-4 and H-2 in acid are non-overlapped within the otherwise overcrowded region, 4.6–0.8 ppm, in the hydrolysate samples (Figure 1) and thereby can be considered reasonable targets for integration. In addition, the residual solvent peak is close to these few int-qHNMR suitable resonances in D_2_O, resulting in integration interference. The only possible countermeasure for making integration more reliable would be to increase acidity to cause a linear downfield shift of the HDO resonance, as discussed above. However, only DCl concentrations >1 M would allow for integration ranges of >30 Hz wide, as suggested by Paudel et al.^50^ As the spectrum of Hyp alone cannot be considered overcrowded, this approach would still only be applicable to the peak patterns of H-4 and H-2, not those of the other four protons of Hyp observed.

Collectively, this highlights the intrinsic limitations of integration in qHNMR: peak patterns with overlapping lines and/or interfering with residual solvent signals, as well as peak patterns fully overlapped with those of other compounds in such complex samples, clearly interfere unfavorably and essentially void integration, as discussed previously by Achanta et al.^25^ For DSS, the situation differs compared to Hyp as, despite the occurrence of ^29^Si satellites, DSS gives rise to an essential singlet that is more straightforward to integrate (but also not very specific because a singlet contains low structural information). Interestingly, and similarly to Hyp, also, the theoretical DSS concentration was underestimated within 1σ for the 0.00–0.99 M DCl solutions, while it was overestimated within 2σ, increasing the acid concentration, 1.5–6.0 M DCl solutions. Both Hyp and DSS’s accuracy decreases as ion concentration increases, even if it is less evident for DSS. While Riemer et al. have used 3-trimethylsilyl-propionate-*d*4 (TSP) as an internal standard despite its low resistance to acid, DSS is more stable and suitable.^20,51,52^

One spectrum for each acidic tested solution was chosen, taking into consideration the lowest FWHM and LQF among the available recordings. These spectra were further processed with MNova, and HifSA profiles were subsequently created using the iterative QMSA process in CT. In this process, the NMR spin parameters are adjusted to minimize the local root-mean-square (LRMS) of the difference between the experimental and the calculated spectra (residual) until they match in every detail. This fine-tuning regards not only the δ values and *J* couplings but also the line widths and relaxation response factors, which correct the intensities for each spin particle according to its individual relaxation, normalizing the spin with the most complete relaxation (the highest intensity) to 1. As this parameter completely correlates with the populations, all response factors were set to 1 and locked for all spins used for quantitation to remove this correlation.^25,40,50^ Population-based quantification of Hyp within CT showed that all the solvents tested were able to return values within 1 σ from the theoretical value and that the variation among samples was random. This also proved that the apparent linear increase in Hyp recovery, obtained via int-qHNMR for all evaluated peak patterns, was an integration artifact, which can be overcome by a QM-assisted qHNMR tool, such as CT.

Nevertheless, the 0.61 M DCl condition was chosen for further method validation. This acidic condition offered the best compromise regarding the residual solvent peak shift for integration, with an int-qHNMR recovery of 99.2% for Hyp and the best capability to tune and match the probe. The ultimate goal was to obtain quantitative data for the hydrolysate samples at <5% error.

### 3.2 qHNMR Method Validation

This part of the study assessed recovery, linearity, including determination of LOQ and LOD, precision, repeatability, and stability of the qHNMR method.

Recovery was evaluated by spiking the turkey hydrolysate sample TC_A (Supporting Information Figure S4), containing low amounts of collagen (<1 w/w %), with low, medium, and high levels of Hyp and Gly, each in triplicate (*n* = 3). Quantification was employed using ERETIC2 on the area of the H-4 and H-2 peak patterns for Hyp and the only singlet available for Gly. Gly was included together with Hyp because it is one of the three amino acids contained in the repetitive unit of collagen that contributes to its characteristic helical structure. This small achiral amino acid was also, and more importantly, included to compare its quantification with Hyp because of the different peak patterns and the different locations with respect to the hydrolysate ^1^H NMR spectrum (Figure 1). A solution containing 2.01 mM DMSO_2_ and 1.67 mM DSS in 0.600 mL of D_2_O served as an external calibrant.

Table 3 indicates that the spiking was precise across the samples, with relative standard deviations (RSD) between 0.30% and 2.28%. However, the recovery accuracy ranged from 80.8% to 93.7%, with the accuracy of Gly being consistently higher than Hyp. This indicated that the Gly singlet, despite being in the middle of an overcrowded region, is a more selective measure of total Gly abundance than the H-4/H-2 multiplet is for Hyp, whose peak patterns belong to two magnetically inequivalent nuclei but, importantly, are in closer proximity to the residual solvent peak. As expected, accuracy was increased by the QM-qHNMR approach, yielding spiking recoveries between 83.4% and 100.9%, with 10% and 1% errors for the low concentration spiking of Hyp and Gly, respectively (Supporting Information Table S5). Notably, recovery accuracy for the low spiking was on average 10% and 6% higher compared to the medium and high spiking for int-qHNMR and QM-qHNMR, respectively. This could potentially be a result of volumetric errors of the pipette which will have a larger impact when dispensing solutions with higher concentrations.

**Table 3:** Results of the recovery test calculated by ERETIC2 using the area of the peak patterns of H-4 and H-2 for Hyp and the sole singlet of Gly. Three different spiking amounts were added to the TC_A sample, both for Hyp and Gly: low, medium, and high spiking (*n* = 3). For each subset, the table reports the amount added, the average recovery, and the relative standard deviation (RSD).

	Recovery test					
	Amount added (mg)		Average recovery (%)		RSD (%)	
Spike	Hyp	Gly	Hyp	Gly	Hyp	Gly
Low	0.24	0.38	92.7	93.7	2.28	1.95
Medium	0.94	1.50	81.8	86.7	1.24	0.30
High	2.36	3.75	80.8	85.7	1.09	1.62

Considering the relative error in Hyp recovery, QM-qHNMR resulted in lower errors than int-qHNMR for the medium and high spiking but higher errors for low spiking. This apparent advantage of int-qHNMR for the low spiking likely reflects baseline-related integration bias affecting low-intensity multiplets. However, the error for Hyp never exceeded the value stated by commercial analytical laboratories using HPLC, 20%, despite the technical imprecision on medium and high spikes. These errors (HPLC and qHNMR) are lower than the one reported for colorimetric methods (10–50%), which are affected by background variation from sample to sample, procedural imprecision such as time, temperature and acid concentration variations, and the interference of other amino acids, which in turn also produce chromogen.^16^ However, despite the improvements due to QM-qHNMR, the error for Hyp is still 10% and has not decreased to 1% as the error for Gly. This is probably due to the different locations of the resonances in the spectrum and their different nature. Gly gives rise to a high S/N ratio singlet that stands out in a relatively uncrowded region of the spectrum. In contrast, Hyp peak patterns are multiplets that have a lower S/N ratio compared to a singlet of equal area. In addition, only H-2/H-4 is outside the highly crowded region, H­(*pro-S*)-3 is partially hidden by the background and all the other Hyp peak patterns are totally hidden by the underlying background. The challenge posed by the undefined background for Hyp quantification is reflected in the uncertainty difference with Gly quantification; future assignment of the underlying peak patterns could improve the accuracy of the iterative QM process and, consequently, Hyp quantification.

Linearity was assessed by spiking (*n* = 2) the other low-collagen hydrolysate sample, TC_B, with different amounts of Hyp and Gly to cover the range of 0.00–15.0 mM for both amino acids. Plotting the absolute integral values against the spike concentrations yielded the linearities with *R* ^2^ values of 0.995 and 0.983 for Hyp (Supporting Information Figure S5B) and Gly (Supporting Information Figure S5A), respectively. This outcome is in line with the known inherently quantitative nature of NMR spectroscopy.^40,42,53,54^ Calculation of LOQ and LOD values following eqs 1 and 2, respectively, gave 3.59 and 1.09 mM for Hyp, and 6.56 and 1.99 mM for Gly, respectively. Considering the corresponding S/N ratios at these concentrations, it is evident that the S/N values for Hyp, 114 for the LOQ and 18 for the LOD, are substantially lower than those for Gly, 1148 for the LOQ and 359 for the LOD. This difference clearly reflects the broader intensity distribution of the H-4/H-2 multiplets compared to the true singlet nature of the H-alpha peak patterns of Gly and the respective spectral region in which they are compared to the background. Overall, eq 1 for the LOQ and eq 2 for the LOD are based on a calibration approach that was developed and is typical for chromatography, which has different principles from NMR, and its translation into S/N, which is traditionally more used for qHNMR, reflects its origin. Gly was not less suited than Hyp for quantification, as demonstrated by the method validation, but less specific due to its singlet nature, as demonstrated by QM-qHNMR. However, expanding this discussion would go beyond the scope of the present study, and the S/N values were in line with qHNMR literature recommendations, suggesting sufficient numbers of scans to achieve S/N values of ≥100–150 to 1000.^42,50^

The precision and repeatability results of the int-qHNMR measurements evaluated as RSD (eq 3) and error % (eq 4) are presented in Figure 5. Testing repeatability in three different days over three different weeks with two independent samples of the same turkey hydrolysate material TC_E, the RSD was ≤1% for Gly and DSS. In comparison, the RSD was only 3.6% for Hyp when quantifying via H-4/H-2 and even lower at 7.9% when integrating the H­(*pro-S*)-3 multiplet. However, such limitations did not come unexpectedly due to the nature of the respective peak patterns. Whereas Gly and DSS both give rise to singlets with a well-defined and narrow range, the peak patterns of the Hyp multiplets cover a much wider range. Accordingly, integration uncertainties increase for such multiplets due to a larger background and variations from integration due to difficulties in consistently correcting for bias and slope manually. Classical integration is particularly vulnerable to baseline imperfections from nearby residual solvent peaks, as in the case for H-4/H-2, or when the target peak pattern lies within a highly crowded region with an increased underlying background, as observed for H­(*pro-S*)-3. Collectively, these factors re-emphasize the inherent limitations of classical int-qHNMR.

**Figure 5:**
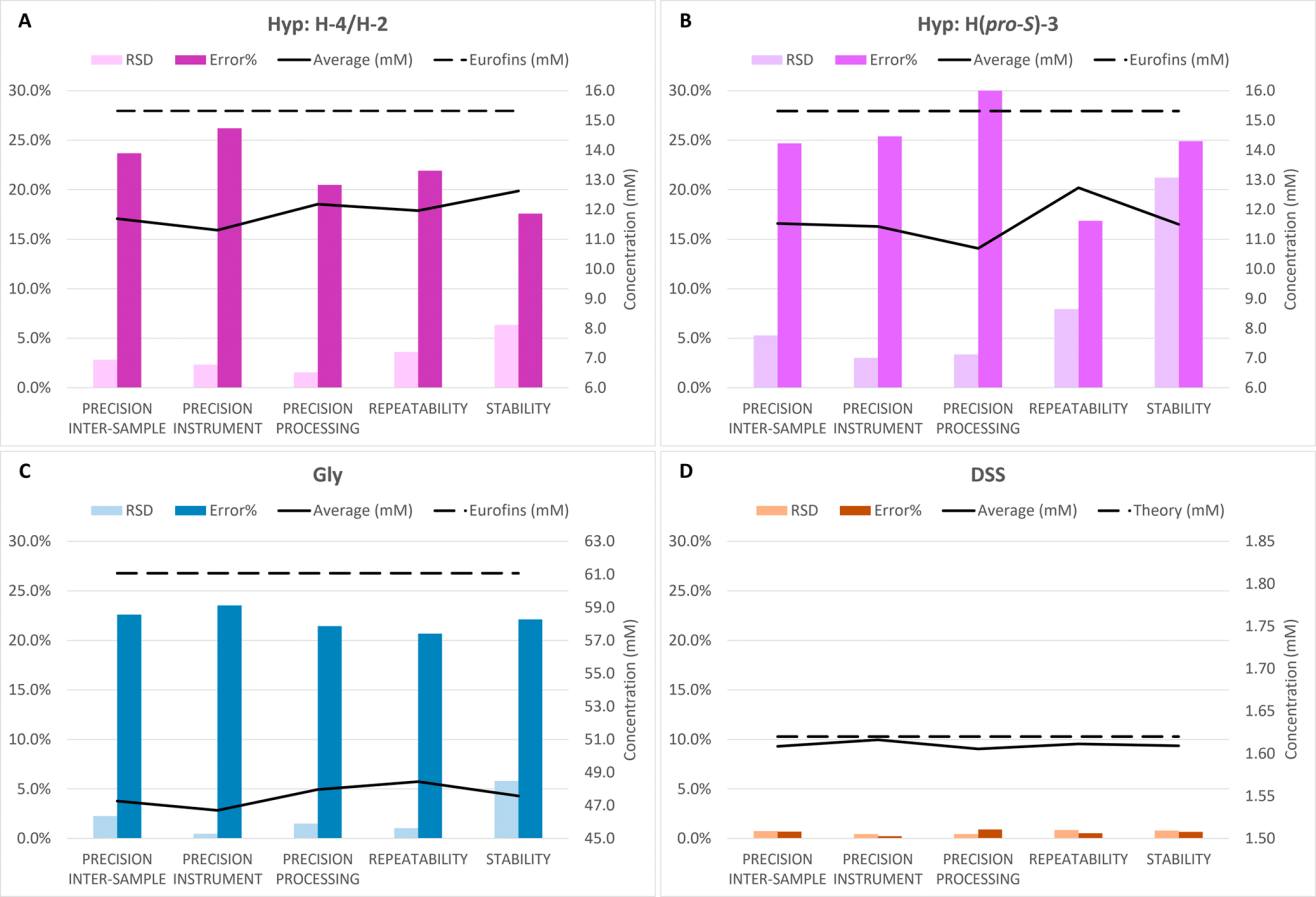
Validation of precision, repeatability, and stability of the int-qHNMR analysis of the hydrolysate samples, TC_E. The precision was divided as intersample (TC_E prepared multiple times the same day; *n* = 6), instrument (multiple injections in the instrument of the same TC_E sample; *n* = 6), and processing (the same TC_E raw spectrum processed multiple times; *n* = 6). The repeatability was evaluated as interday variability (TC_E sample prepared in biological duplicates three times in three different weeks; *n* = 6). The stability was evaluated over 2 months (time series injections of the same TC_E sample; *n* = 12). The following components were analyzed: hydroxyproline (Hyp {violet}; multiplet H-4/H-2 [panel A] and multiplet H­(*pro-S*)-3 [panel B]), glycine (Gly {blue}; singlet [panel C]), and DSS ({brown} singlet at 0.000 ppm [panel D]). For each validation parameter, the relative standard deviation (RSD) and relative errors (error %) of the recovery are reported in percentages as a bar chart (light and dark colors, respectively), while the average of the concentration calculated with ERETIC2 and the values reported by Eurofins (amino acid analysis according to ISO 13903:2005, EU 152/2009 (F)) for the same samples are reported as a line chart (solid and dashed lines, respectively).

Analysis of intersample precision, performed using six independent TC_E samples prepared the same day, generally showed lower RSD values than those observed in the repeatability test, with the exception of Gly. Moreover, the results indicate that the largest contribution to precision variability does not arise from sample weighing or pipetting (precision intersample in Figure 5), which otherwise are major sources of uncertainty in accuracy, but rather from the processing of the spectra (precision processing in Figure 5), in particular, integration. The contribution to precision (RSD) of the processing was higher than the contribution of the instrument (precision instrument in Figure 5) for H­(*pro-S*)-3, Gly, and DSS. Indeed, variability introduced during spectral processing (often operator-dependent) constitutes a substantial portion of the overall achievable precision (intersample + instrument + processing), highlighting the challenges associated with analyzing such complex spectra.

Based on the data presented in Figure 5, it is possible to conclude that the recovery is again around 80% both for Gly and Hyp H-4/H-2 but higher (≥99%) for DSS. This makes DSS a suitable internal standard and calibrant and confirms its stability in acid.

To confirm sample stability, the same TC_E sample was analyzed at 0.5, 2.0, 3.0, 5.5, 10.5, 20, 40, and 72 h as well as after 1, 2, 3, and 9 weeks (*n* = 12). While the RSD was higher than that in the previous cases, at approximately 6.1% for both Hyp (based on H-4/H-2) and Gly, it remained constant for DSS at 0.80%. However, all variations were within 2σ from the average, except those for Hyp H-4/H-2 at 9 weeks, which were still inside 3σ (Supporting Information Figure S6). This may be a result of variation caused by the integration process, with a high error contribution stemming from the bias and slope correction of the integrals. In comparison to int-qHNMR, QM-qHNMR-based stability data (Supporting Information Figure S7) showed higher consistency with Hyp values all falling within 1σ, despite the recognized shimming artifacts of the 40 h spectrum. The Gly QM-qHNMR stability values coincided with those from int-qHNMR, which was expected due to the singlet nature of the integrated/QM-fitted peak pattern.

A closer analysis revealed that the spectra could be grouped by shimming quality when applying resolution-enhancing spectral processing (Lorentzian–Gaussian window function; Supporting Information Figure S8), explaining the outliers such as the 40 h spectrum and RSD. Comparing the RSD of QM-qHNMR with int-qHNMR, they were 3.5% and 6.4% for Hyp and 7.5% and 5.8% for Gly, respectively. On one side, this highlighted the robustness of QMSA because the Hyp can be calculated on a statistical basis due to multiple peak patterns available. On the other side, this showed the weakness of analyzing a singlet such as that from Gly in an overcrowded region, where no other peak patterns can be used to increase robustness. However, as long as the investigated samples did not undergo any visible alteration, such as precipitation or evaporation, during the 2 month test period, they were considered stable. This was confirmed by the observation that all recorded measurements fell within 1–2σ from the average (Figures 5, Supporting Information Figure S6 and S7).

Finally, additional data analysis revealed a relationship between the chemical shift of the HDO peak, which is a function of acidity (see discussion above), and the time between the sample preparation and the sample analysis (Supporting Information Figure S9). Within 2 months, the HDO peak shifted 4.5 ppb downfield from 5.1105 to 5.1150 ppm. This small peak shift, corresponding to a minimal 0.0006 pH difference according to Figure S2, can potentially be attributed to solvent evaporation, occurring despite the capping and parafilm sealing of the tube. This was considered a minor contributor among the variables considered for the stability of the sample but a factor to consider in case of long-term storage of samples (e.g., sample shipping). This finding underlined that compared to simpler types of samples, it is important to analyze hydrolysate samples within a certain time frame to allow their comparison, for example, 1 week. The background makes even small variations in volume and ion species causes of variability, up to a general 5% error. This additional source of uncertainty is undesired when high accuracy is pursued.

Overall, the presented validation of int- and QM-based qHNMR methods for collagen amino acid determination adds valuable insights to the prior findings by Riemer et al. The present outcomes demonstrate that qHNMR, in general, has more than adequate accuracy, precision, and reproducibility and compares favorably to other available analytical techniques such as HPLC or colorimetry. Thus, qHNMR is ready to be adopted not only as a powerful alternative for quantitation but also holds other advantages such as providing simultaneous information about the structural integrity and possible alterations in the sample. Its ability to be expanded to the detection and quantification of multiple targets (e.g., other amino acids) adds to the appeal of qHNMR and makes it superior to other currently used methods.

### 3.3 Comparison of Different qHNMR Methods

The amount of Hyp in turkey hydrolysates was determined by two main techniques: standardized HPLC analysis performed by a commercial analytical laboratory and qHNMR analysis performed in-house. For qHNMR analysis, three different methods of deriving quantitative measures from the NMR data were used: integration and external calibration (EC) using the Bruker ERETIC2 method, integration and combined external and internal calibration (ECIC) with manual calculation by spreadsheet (see Supporting Information Section S3), and QMSA/HifSA-based analysis, also with ECIC, using the populations as measures. The results of this exploratory comparison are presented in Figure 6.

**Figure 6:**
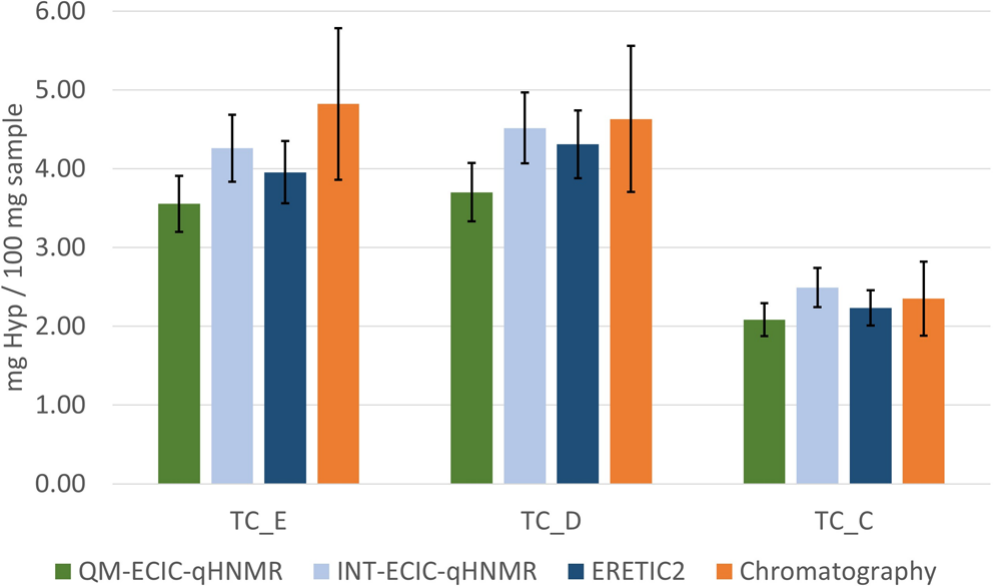
Comparison of four quantification methods for hydroxyproline (Hyp) in protein hydrolysates: HPLC analysis performed by the commercial analytical laboratory Eurofins (orange); qHNMR analysis with integrals and ERETIC2 as implemented into Bruker TopSpin (dark blue); int-qHNMR with ECIC calculations (light blue); QM-qHNMR performed within Cosmic Truth (CT) and ECIC (green). An in-house prepared calibrant containing 2.01 mM DMSO_2_ and 1.67 mM DSS in D_2_O was used as EC for ERETIC2 and as ECIC for spreadsheet-based manual calculations (IC: DSS, EC: DMSO_2_). Three turkey hydrolysates were compared: TC_E (*n* = 1), TC_D (*n* = 1), and TC_C (*n* = 1). According to the industry standard HPLC analysis, the samples contained 4.82%, 4.63%, and 2.35% Hyp (w/w %), respectively. The error bars reported for the HPLC method correspond to 20% as declared by the commercial analytical laboratory, while the error bars reported for the qHNMR methods correspond to 10% as described in the Method Validation section.

Surprisingly, the amount of Hyp determined by int-ECIC-qHNMR was consistently higher than that obtained by ERETIC2, despite both methods using the same integral values. The Hyp amount was 6.5% and 6.0% higher for TC_E and TC_D, respectively, while it was 12% higher for TC_C. This can plausibly be attributed to two factors: (a) the difference in the corrections applied by the ERETIC2 algorithm using the constant *k* in eq 5: this approach takes into consideration differences in RG values, which represent a semilinear function, and *T*_1_;^55,56^ (b) the nature of the correction applied in ECIC quantitation in which the IC, in this case the DSS, amounts to most of the differences in acquisition parameters and tubes. Our study applied DSS rather than the residual solvent signal as IC for two main reasons: (i) because of the uncertainty of the final volume in the NMR tube due to the volatility of the acid and the filtration step; (ii) because the samples and the EC, despite being in the same solvent (D_2_O), had different ionic strengths due to the different DCl concentrations and varying amounts of salt in the hydrolysates.

Compared with both int-qHNMR approaches, the QM-qHNMR method yielded lower concentration values for the same qHNMR spectra. This was already observed by Choules et al., whose purity determinations by QM-qHNMR returned consistently lower percentages than via integration.^27^ The earlier explanations for these observations also resonate for this study: integration uses peak patterns that are considered non-overlapping and is restricted to the apparently “clean” regions of the spectrum. However, plain integration has no methodological means of verifying its specificity, especially in the absence of a control peak, which is a ubiquitous limitation for chemically complex samples, such as hydrolysates. In contrast, QM is anchored in all spin parameters, and their correlations are used in the analysis of a target analyte. Thus, it can overcome the challenges of highly overlapped regions as long as spin particles belonging to the same molecule resonate in other parts of the spectrum. Interpreted from a different point of view, QM-qHNMR analysis uses spectral parameters linked to the analyte structure and according to the same quantum mechanical principles that represent the foundation of NMR spectroscopy altogether. QM-qHNMR quantitation involves optimization by an iterative process of fitting, aimed at producing the most accurate replica of the experimental spectrum possible. Therefore, it is generally regarded as a more critical and accurate quantitation method than peak-fitting deconvolution and classical integration because it typically yields values closer to the true concentrations.^25,27,57,58^

In general, the current industry standard HPLC method reported a higher amount of Hyp for all samples, but the NMR values remain in the range of the (relatively broad) confidence interval of the HPLC method in almost all cases. These observations are based on a small data set (*n* = 3) and would benefit from a broader future comparison to achieve a relevant statistical significance. However, qNMR is a direct quantification method that does not rely on derivatization, analysis-specific reference standards, or chromatographic separation, resulting in a simpler protocol and reduced quantification uncertainty compared to HPLC. qHNMR is fast and highly informative because it is structure-related, making it particularly advantageous for the analysis of uncharacterized samples with complex matrixes. By contrast, the HPLC method requires careful assessment of potential matrix effects. The comparison across three different sample types highlights the differences between HPLC and QM-qHNMR and their potentially broader impact. While the Hyp content ranked TC_E > TC_D > TC_C based on the HPLC analyses, it became TC_D > TC_E > TC_C with QM-qHNMR. Interestingly, the Hyp amount determined by QM-qHNMR is better in line with the amount of ash in the samples (Table 1). This can be explained by the EPH process: when bulk collagen is hydrolyzed into peptides during the EPH process, it converts not only the collagen from the collagenous fibers in the cartilage but also that from the bone extracellular matrix (ECM).^59^ Therefore, the major inorganic component of the bone’s ECM, hydroxyapatite, is also released into the solution along with the collagen peptides.^60,61^

In summary, this study established qHNMR methods for collagen quantification in protein hydrolysates through the quantification of its characteristic major amino acid, Hyp. Solvent ionic strengths were explored, and their challenges and advantages were analyzed. The use of high concentrations of acid as a shifting agent to allow int-qHNMR was found to have inferior accuracy to the QM-qHNMR by HifSA. Moreover, in the balance between shorter *T*_1_ and consequent acquisition parameters on one side and T/M of the probe on the other side, acid has no practical advantage for the qHNMR analysis. However, a low concentration of acid was maintained because it resulted in optimal T/M conditions. In terms of qHNMR methodology, the study showed that QM-qHNMR can resolve peak overlap for all the tested solvents, representing a major advantage over int-qHNMR. Method validation demonstrated that qHNMR has favorable specificity, sufficiently high sensitivity, the ability to overcome background interference, and adequate repeatability.

Finally, it should be kept in mind that HifSA profiles are highly definitive and transferable within and between laboratories and different instruments. Accordingly, QM-qNMR has the potential to simplify the broader adoption of qNMR methods in chemical analyses, which to date have been dominated by HPLC and colorimetric assays, despite their known higher uncertainties. As demonstrated, QM-qHNMR can provide lower uncertainties in the quantification of Hyp (10%) compared to the established techniques (e.g., 20% for HPLC) because of its inherent higher precision and accuracy. In addition, it is a more universal method that is not affected by the background to the same extent as the other classical methods are, and potentially, the same QM-qHNMR workflow can be applied to spectra from samples with different matrixes. Moreover, QM-qHNMR is a fully scalable technology that ensures its validity across various magnetic field strengths. This enables the adoption of low-field NMR, such as within industrial settings and as part of the production of peptides from food side streams by EPH. This study represents a first step forward toward the adoption of qNMR as a faster, less demanding, and more accurate multitarget amino acid analysis for complex protein hydrolysates.

## Supplementary Material



## Notes

Additional raw material details; experimental setup including the code for running the NMR spectrometer and details regarding acquisition, processing, and quantitative calculations; and additional results including ^1^H NMR spectra of hydroxyproline and hydrolysates and tables and plots of ^1^H NMR parameters correlated (PDF)
