# Supplementary material for: NMR-Based Quantification of Collagen Content in Protein Hydrolysates
Source: J Agric Food Chem. 2026 Feb 9;74(6):5627–39. doi: 10.1021/acs.jafc.5c13447 (PMC12921863; doi:10.1021/acs.jafc.5c13447)
Supplement: Supplementary file 1 [file jf5c13447_si_001.pdf]

Supporting Information

NMR-based Quantification of Collagen Content in Protein Hydrolysates

Greta Nardini[a,*], Kristian Hovde Liland[b], Guido F. Pauli[c], Sileshi Gizachew Wubshet[d], Alistair L. Wilkins[e], Frode Rise[f], Matthias Niemitz[g], Nils Kristian Afseth[d], and Kenneth Aase Kristoffersen[a]

a) Faculty of Chemistry, Biotechnology and Food Science, Norwegian University of Life Sciences, P.O. Box 5003, NO-1432 Ås, Norway. b) Faculty of Science and Technology, Norwegian University of Life Science, P.O. Box 5003, NO-1433, Ås, Norway. c) Pharmacognosy Institute & Department of Pharmaceutical Sciences, Retzky College of Pharmacy, University of Illinois Chicago, IL 60612, United States of America. d) Nofima - Norwegian Institute of Food, Fisheries and Aquaculture Research, P.O. Box 210, NO-1431, Ås, Norway. e) Chemistry Department, The University of Waikato, Hamilton 3240, New Zealand. f) University of Oslo, Department of Chemistry, P.O. Box 1033, Blindern, NO-0315, Oslo, Norway. *g) NMR Solutions Ltd., 70110 Kuopio, Finland.*

**Corresponding author: Greta Nardini:* greta.nardini@nmbu.no

Table of Contents

1. Samples and raw materials........S-3

Overview of the origin of the turkey hydrolysates samples – Table S-1........S-3

2. Instrument adjustments........S-3

Phyton script........S-3

3. ECIC calculations........S-4

Samples details........S-4

NMR parameters........S-4

$DMSO_2$ and hydroxyproline ^{1}HNMR spectra with integrals and populations........S-5

Step by step calculations........S-5

4. Effect of different solvent acidity........S-6

Deshielding effect in Hydroxyproline ^{1}HNMR spectra – Figure S-1........S-6

Chemical shifts (δ) for hydroxyproline Hs and water peak – Table S-2........S-7

Correlation between water peak shift and acid concentration – Figure S-2........S-7

Coupling constants (J) for hydroxyproline – Table S-3........S-8

Tuning and matching curves – Figure S-3........S-9

Minimum values for tuning and matching curves – Table S-4........S-9

5. Method validation........S-10

Turkey hydrolysates ^{1}HNMR spectra with spiking – Figure S-4........S-10

Recovery test by QM-qHNMR – Table S-5........S-11

Calibration curves for LOQ and LOD of glycine and hydroxyproline – Figure S-5........S-11

Stability of turkey hydrolysate by int-qHNMR – Figure S-6........S-13

Stability of turkey hydrolysate by QM-qHNMR – Figure S-7........S-14

Shimming quality of the 1HNMR spectra in the stability test – Figure S-8........S-14

Correlation between water peak shift and time – Figure S-9........S-15

Bibliography........S-15

1. Samples and raw materials

Table S-1: Overview of the origin of the turkey hydrolysate samples. For each sample used in this study, it provides the original name used in the previous study by Kristoffersen et al. and Lindberg et al., the raw material, and the enzyme used to produce the hydrolysate with the respective time of hydrolysis.[1, 2] The combination of hydrolysis time and enzyme explains the collagen amount is included in the main text.

Sample name	Original paper sample name	Raw material	Enzyme	Hydrolysis time (min)
TC_A	TCB	Turkey carcasses	Bromelain	5
TC_B	TCE	Turkey carcasses	Endocut-02	15
TC_C	MDTR_Fl	Mechanical deboned turkey residue	Flavourzyme	80
TC_D	MDTR_Co	Mechanical deboned turkey residue	Corolase 2TS	80
TC_E	MDTR_Al	Mechanical deboned turkey residue	Alcalase 2.4 L	80

2. Instrument adjustments

Instrument adjustments were performed as Phyton script to run the instrument in semi-manual mode: locking, waiting, shimming, tuning and matching.

XCMD ("lock solvent_name") #lock solvent

SLEEP (300) #wait 5 min

XCMD ("rsh solvent_name") #read the shim file saved for the solvent

XCMD ("topshim z6off 1d optsolvent coil=1H tuneboff tuneaoff") #1st run with classical Topshim command

XCMD ("topshim tunebxyz convcomp tunea") #2nd run with high conductivity sample optimized command

XCMD ("topshim z6off 1d optsolvent coil=1H tuneboff tuneaoff") #3rd run with classical Topshim command

XCMD ("atma exact storewobb xxx") #automatic tuning and matching with wobb curve stored in specified procno

3. ECIC calculations

The sample with 13.0 mM of hydroxyproline (Hyp; 12.9 mM corrected for declared purity) and 1.62 mM DSS in 600 µL of 0.61 M DCl in a 5-mm NMR tube from the 2.2.1. Solvent study is used here as example for illustrating the ECIC calculations. The EC sample contained 2.01 mM $DMSO_2$ and 1.67 mM DSS in 600 µL D_2O, as described in the main text. The spectra were acquired and processed as described in 2.5. qNMR experiments.

Samples details:

Target analyte = Hyp (t); Internal Calibrant in analyte sample = DSS (IC-t)

External Calibrant = $DMSO_2$ (EC); Internal Calibrant in EC = DSS (IC(EC))

NMR parameters:

Instrument:	Bruker 400 MHz 5 mm broadband probe (BBO)	
Pulse Program:	zg (90° pulse)	
Sample Temperature:	298 K	
Data Points:	64K	
Acquisition Time (AQ):	4.09 s	
Spectral Window (SW):	8,013 Hz	
Transmitter Offset:	4.427 ppm	
Dummy Scans (DS):	2	
Scans (NS):	16	
90° pulse (P_1):	9.78 µs ($DMSO_2$);	14.57 µs (Hyp)
Relaxation delay (D_1):	90 s ($DMSO_2$);	15 s (Hyp)
Receiver gain (RG):	64 ($DMSO_2$);	57 (Hyp)
Processing:	exponential = -0.3 Hz + Gaussian = 0.3 Hz	
Zero-filling:	128K	
Phasing:	automatic	
Baseline Correction:	multipoint	

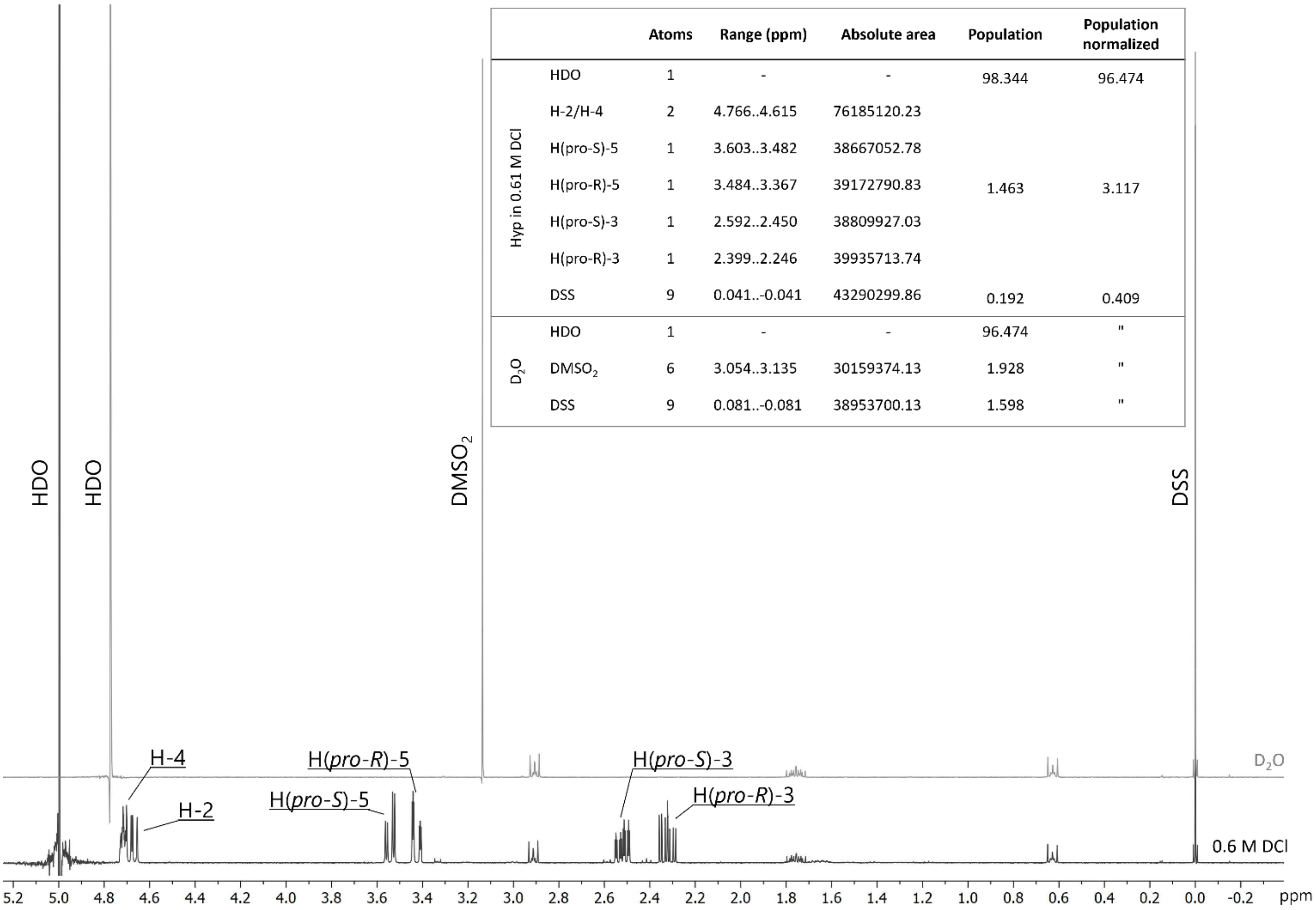

		Atoms	Range (ppm)	Absolute area	Population	Population normalized
Hyp in 0.61 M DCl	HDO	1	-	-	98.344	96.474
	H-2/H-4	2	4.766..4.615	76185120.23		
	H(pro-S)-5	1	3.603..3.482	38667052.78		
	H(pro-R)-5	1	3.484..3.367	39172790.83	1.463	3.117
	H(pro-S)-3	1	2.592..2.450	38809927.03		
	H(pro-R)-3	1	2.399..2.246	39935713.74		
	DSS	9	0.041..-0.041	43290299.86	0.192	0.409
D_2O	HDO	1	-	-	96.474	"
	$DMSO_2$	6	3.054..3.135	30159374.13	1.928	"
	DSS	9	0.081..-0.081	38953700.13	1.598	"

A. Molecular Weight: MW_t = 131.13 g/mol | MW_{IC} = 218.32 g/mol | MW_{EC} = 94.13 g/mol

B. Purity: P_{EC} = 99.96%

C. Mass and volume: m_t = 1.02 mg | m_{EC} = 0.114 mg; V_t = 0.600 mL | V_{EC} = 0.600 mL

D. Average integral per 1H / Population normalized:

a. Int_t = 38935608.90 | int_{EC} = 5026562.35 | $int_{IC\text{-}t}$ = 4824037.77 | $int_{IC(EC)}$ = 4320087.48

b. Pop_t = 3.117 | Pop_{EC} = 1.928 | Pop_{IC} = 0.409 | $Pop_{IC(EC)}$ = 1.598

E. Conc calculation: $P(\%) \cdot \frac{m}{V}$

a. Integrals: $Conc_t = \frac{Int_t \cdot Mw_t \cdot m_R^*}{Int_R \cdot Mw_R \cdot m_t} \cdot P_R \cdot \left(\frac{m_t}{V_t}\right)$ = 1.83 mg/mL → 14.0 mM

b. Population: $Conc_t = \frac{Pop_t \cdot Mw_t \cdot m_R^*}{Pop_R \cdot Mw_R \cdot m_t} \cdot P_R \cdot \left(\frac{m_t}{V_t}\right)$ = 1.67 mg/mL → 12.7 mM

*the reference (R) was the IC-t corrected for IC(EC) that was calculated according to EC. All the calculations and full explanation can be found at http://qnmr.gfpweb.com/qnmr/qnmrcalculations/ec.html.[3]

4. Effect of different solvent acidity

Figure S-1: Deshielding effect in the hydroxyproline (Hyp) ^{1}HNMR spectrum: all the resonances shift due to acid. Stacked view of the Hyp spectra in 0.00, 0.50, 0.61, 0.99, 1.5, 3.0, 6.0 M DCl. Figure A shows the full ^{1}HNMR spectrum, 0.0 to 7.5 ppm; figure B zooms in the region 2.1 to 4.9 ppm.

A

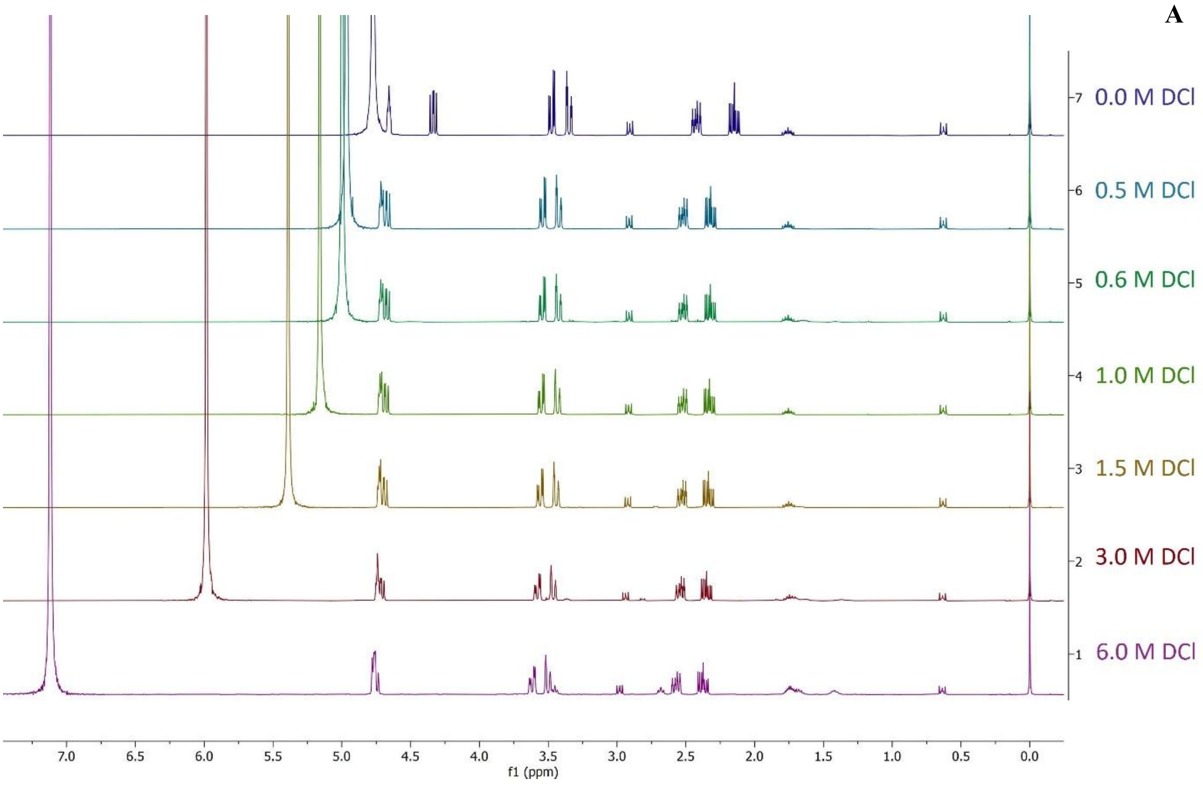

B

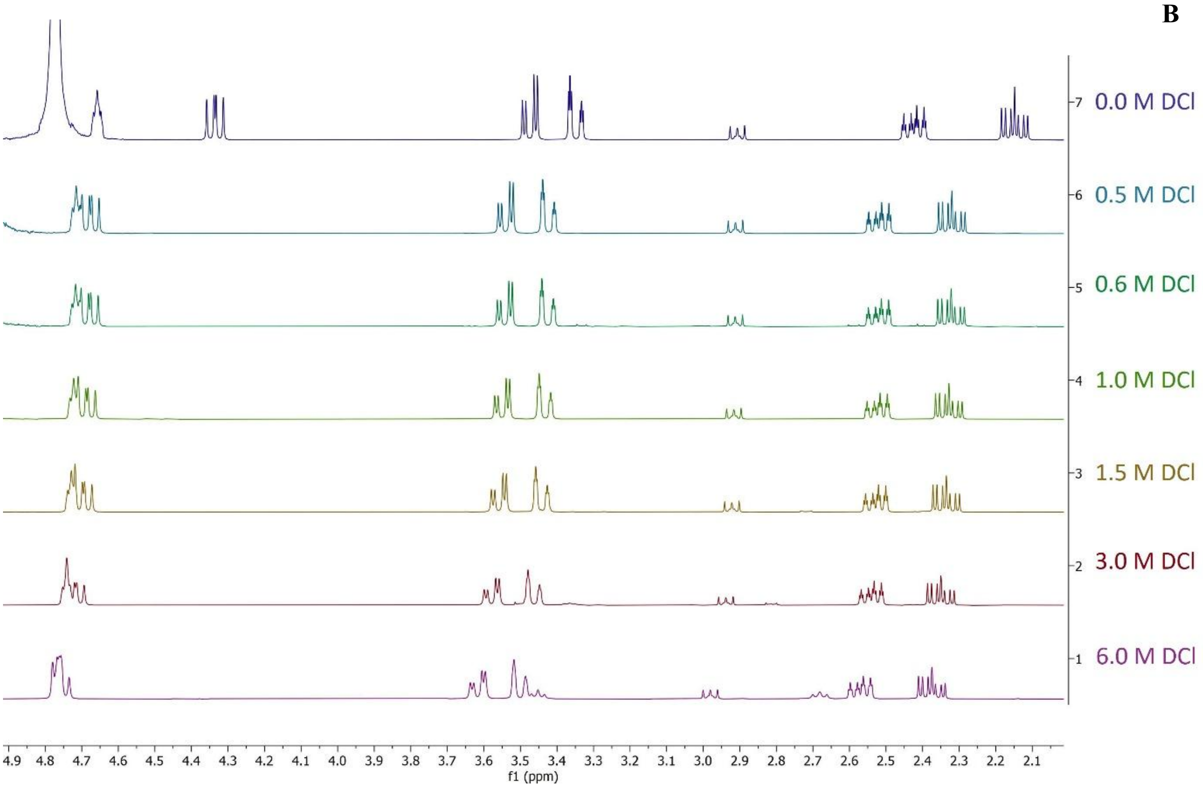

Table S-2: Chemical shifts (δ) observed for all the hydroxyproline (Hyp) protons and the residual water peak (HDO). The values are presented for all the acidic solutions: 0.00, 0.50, 0.61, 0.99, 1.5, 3.0, 6.0 M DCl.

	**Chemical shift (δ, ppm)**						
**Atom name**	0.0 M DCl	0.5 M DCl	0.6 M DCl	1.0 M DCl	1.5 M DCl	3.0 M DCl	6.0 M DCl
HDO	4.770	4.960	5.000	5.160	5.390	5.980	7.120
H-4	4.658	4.715	4.717	4.722	4.728	4.742	4.768
H-2	4.335	4.676	4.678	4.686	4.695	4.716	4.758
H(*pro-S*)-5	3.472	3.538	3.541	3.548	3.557	3.576	3.614
H(*pro-R*)-5	3.351	3.425	3.428	3.435	3.445	3.466	3.504
H(*pro-S*)-3	2.423	2.518	2.519	2.522	2.527	2.539	2.569
H(*pro-R*)-3	2.150	2.322	2.324	2.330	2.337	2.352	2.377

Figure S-2: Linear correlation (R^2 = 0.9995) between the distance of the water residue peak (HDO) from the DSS reference peak (0.000 ppm) and the acid concentration in solution. The HDO chemical shift is presented for all the acidic solutions: 0.00, 0.50, 0.61, 0.99, 1.5, 3.0, 6.0 M DCl.

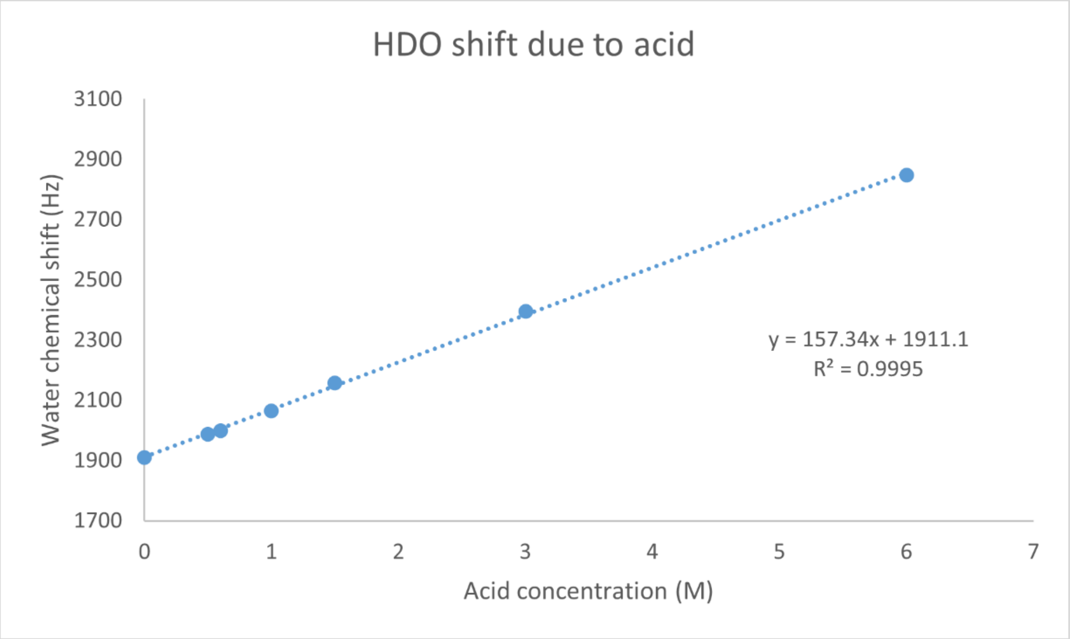

Table S-3: Coupling constant (J) values obtained by HifSA of all the hydroxyproline (Hyp) protons. The values are presented for all the acidic solutions: 0.00, 0.50, 0.61, 0.99, 1.5, 3.0, 6.0 M DCl.

Type	Coupling constant (J, Hz)						
	0.0 M DCl	0.5 M DCl	0.6 M DCl	1.0 M DCl	1.5 M DCl	3.0 M DCl	6.0 M DCl
4J H(*pro-S*)-5 – H(*pro-R*)-3	-0.479	-0.467	-0.464	-0.471	-0.453	-0.441	-0.405
4J H(*pro-R*)-5 – H(*pro-S*)-3	2.073	2.038	2.035	2.031	2.032	2.027	2.035
4J H-4 – H-2	-0.682	-0.687	-0.696	-0.699	-0.697	-0.712	-0.658
3J H-2 – H(*pro-S*)-3	7.966	7.920	7.921	7.924	7.926	7.919	7.915
3J H-2 – H(*pro-R*)-3	10.227	10.497	10.491	10.499	10.503	10.520	10.571
3J H-4 – H(*pro-S*)-3	1.687	1.507	1.513	1.517	1.519	1.521	1.508
3J H-4 – H(*pro-R*)-3	4.362	4.386	4.390	4.386	4.393	4.393	4.393
3J H-4 – H(*pro-S*)-5	3.825	3.827	3.833	3.832	3.828	3.827	3.844
3J H-4 – H(*pro-R*)-5	1.368	1.228	1.235	1.231	1.234	1.229	1.229
2J H(*pro-R*)-3 – H(*pro-S*)-3	-14.105	-14.154	-14.155	-14.157	-14.156	-14.160	-14.211
2J H(*pro-R*)-5 – H(*pro-S*)-5	-12.614	-12.634	-12.638	-12.643	-12.646	-12.661	-12.726

Figure S-3: Tuning and matching (T/M) curve of the probe for all the acidic solutions: 0.00, 0.50, 0.61, 0.99, 1.5, 3.0, 6.0 M DCl (bottom-up). The minimum of the curve increases increasing the acid concentration. The y-axis (Efficiency of match to 50Ω) is an arbitrary axis ([abs.] intensity), it is not calibrated but equal for all the experiments and it shows the amount of power reflected as a function of frequency.

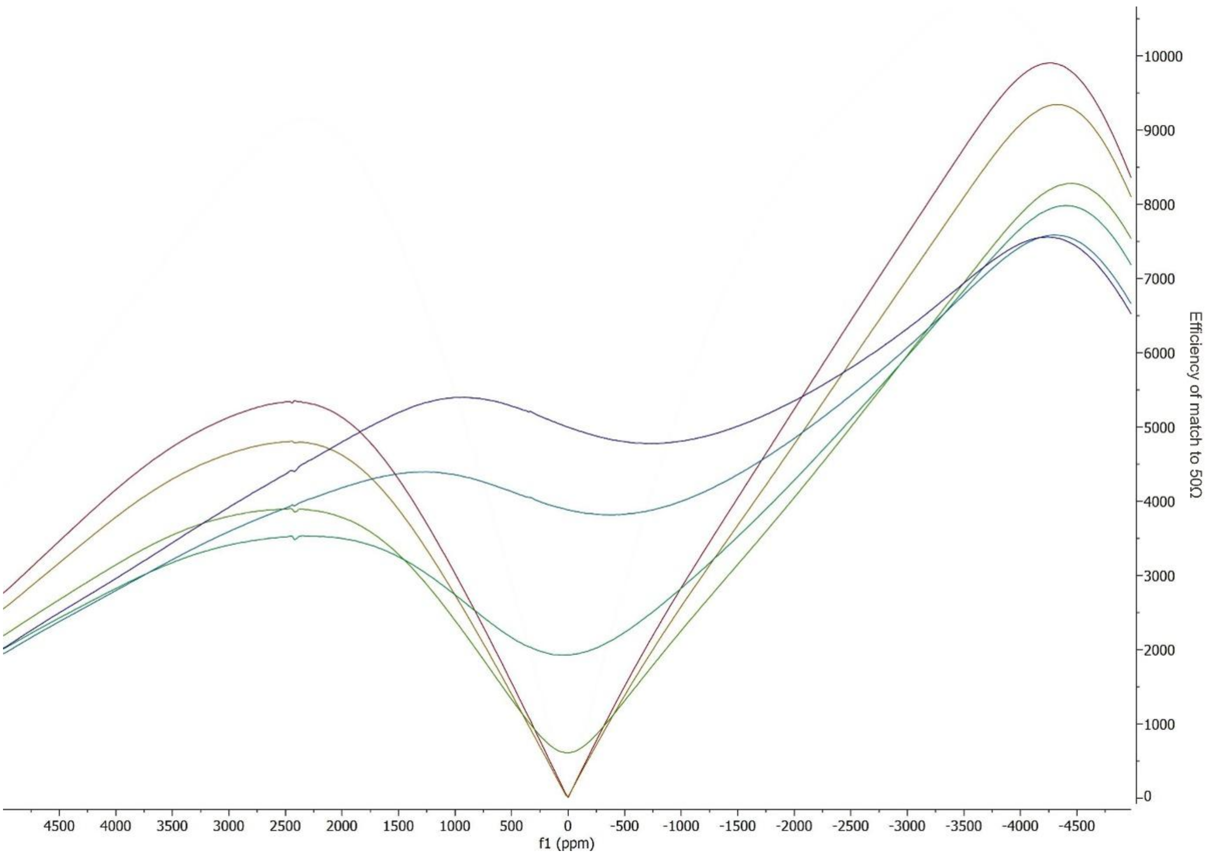

Table S-4: Corresponding minimum values of the tuning and matching (T/M) curve for each acidic solution: 0.00, 0.50, 0.61, 0.99, 1.5, 3.0, 6.0 M DCl. The minima are reported as absolute intensity.

Acid concentration	T/M absolute intensity
0.0 M DCl	90
0.5 M DCl	54
0.6 M DCl	37
1.0 M DCl	615
1.5 M DCl	1930
3.0 M DCl	3817
6.0 M DCl	4780

5. Method validation

Figure S-4: Turkey hydrolysate TC_A qHNMR spectra (400 MHz; 0.00-5.4 ppm) spiked with low, mid and high levels of hydroxyproline (Hyp) and glycine (Gly) is presented in Figure A. The "*" highlight the peak patterns of interest: H-4/H-2 and H(*pro-S*)-3/H(*pro-R*)-3 for Hyp which are outside and within the overcrowded region, respectively; the only available singlet for Gly which is as well inside the overcrowded region of the hydrolysate spectrum. Figure B zooms in the Gly singlet at 3.9 ppm. Figure C zooms in the Hyp multiplet H(*pro-S*)-3 at 2.4 ppm and H(*pro-R*)-3 at 2.3 ppm. Figure D zooms in the Hyp multiplet H-4/H-2 at 4.7 ppm. Each spiking was performed in triplicate (n=3), but only one spectrum per each is shown in the figures for graphical clarity.

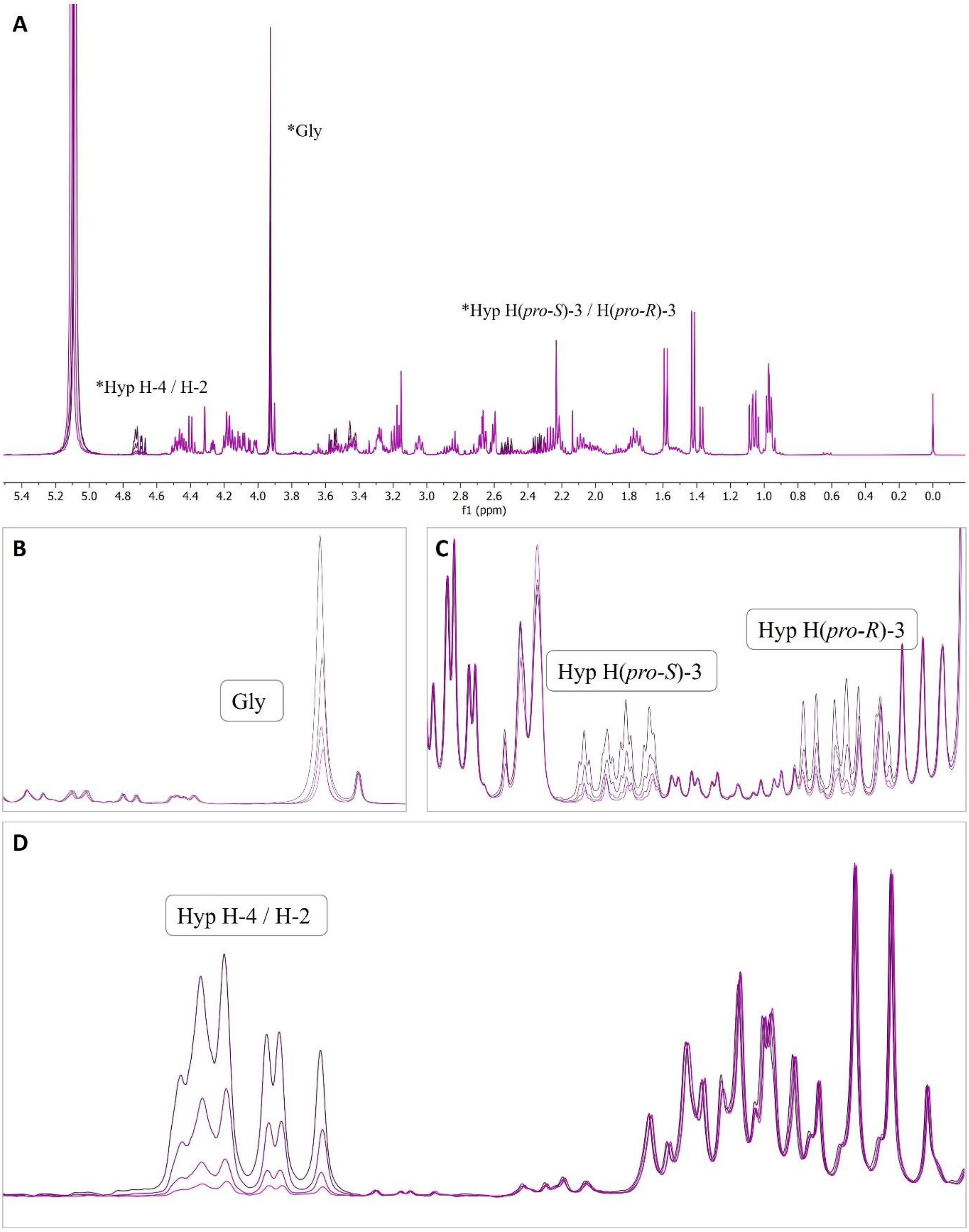

Table S-5: Results of the recovery test calculated by quantum mechanical (QM)-qNMR approach using Cosmic Truth (CT). The same raw spectra used for Table-3 were used here: TC_A sample spiked with low, medium and high amount of hydroxyproline (Hyp) and glycine (Gly) (n=3). For each subset, the table reports the amount added, the average recovery, and the relative standard deviation (RSD).

	RECOVERY TEST					
**Spike**	**Amount added (mg)**		**Average recovery (%)**		**RSD (%)**	
	Hyp	Gly	Hyp	Gly	Hyp	Gly
Low	0.24	0.38	90.7	100.9	2.92	1.35
Medium	0.94	1.50	85.1	95.8	1.18	1.25
High	2.36	3.75	83.4	95.3	1.16	0.60

Figure S-5:Calibration curves for glycine (Gly, [panel A]) and hydroxyproline (Hyp, peak patterns H-2/H-4, [panel B]) in which the absolute integral values are plotted against the spiked concentrations. Panel A and B proved the linearity of qHNMR inherent to its nature. Correlation of the LOQ and LOD concentration values with their respective signal-to-noise (S/N) values for Gly (Panel C) and Hyp (Panel D). The concentration reported in the X axis of panel C and D are the concentration found: spiked amount + background from the sample.

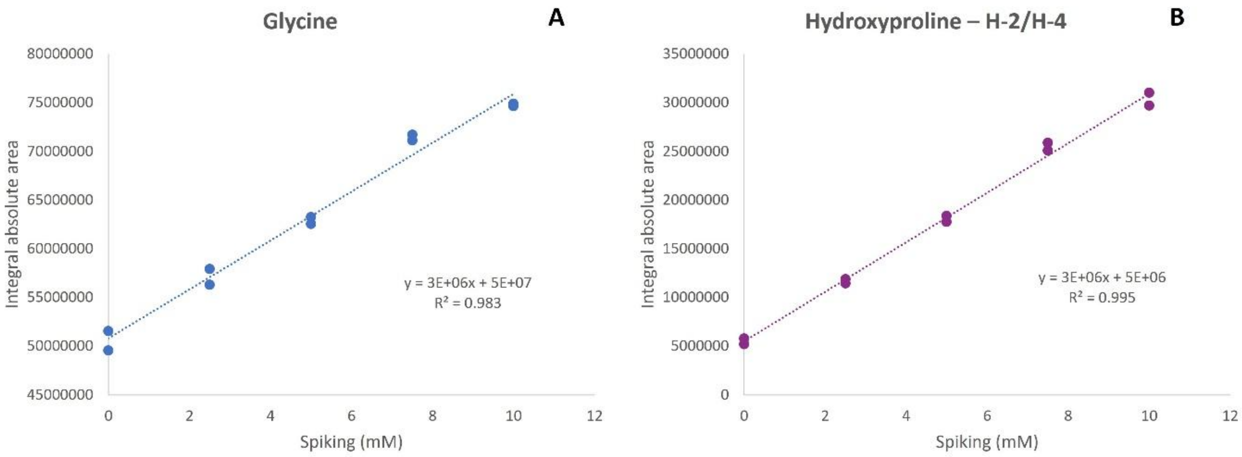

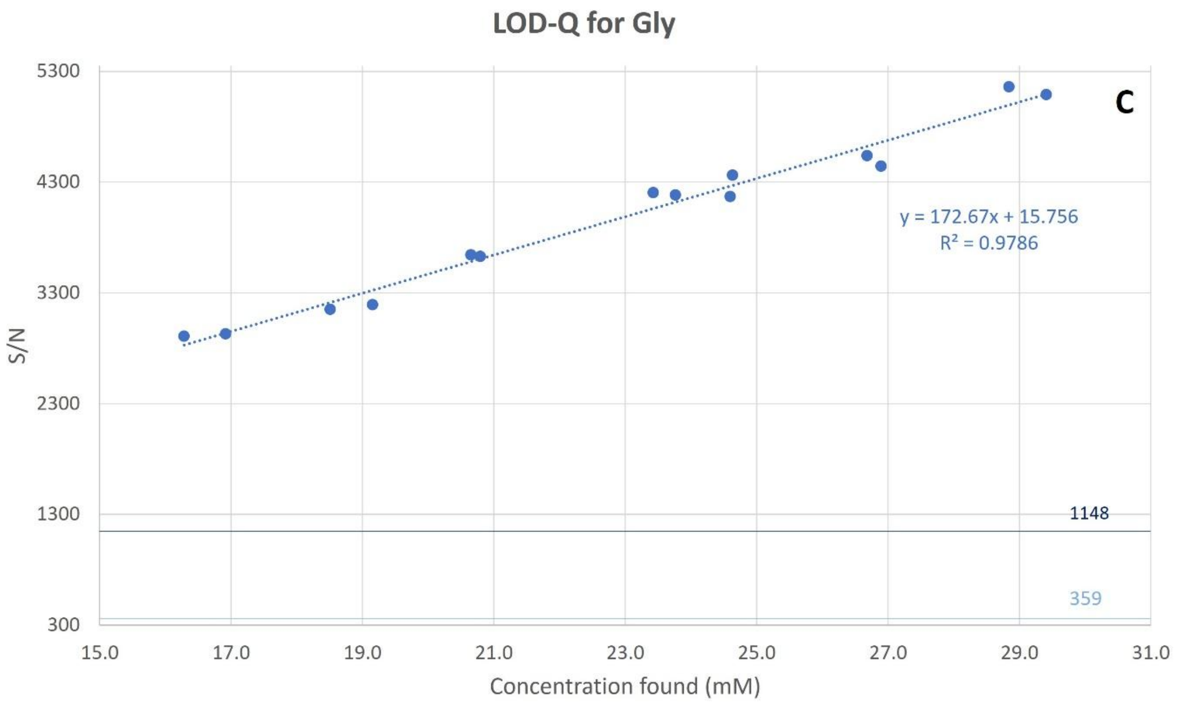
LOD-Q for Gly
C
y = 172.67x + 15.756
R² = 0.9786
1148
359
S/N
Concentration found (mM)
5300
4300
3300
2300
1300
300
15.0
17.0
19.0
21.0
23.0
25.0
27.0
29.0
31.0

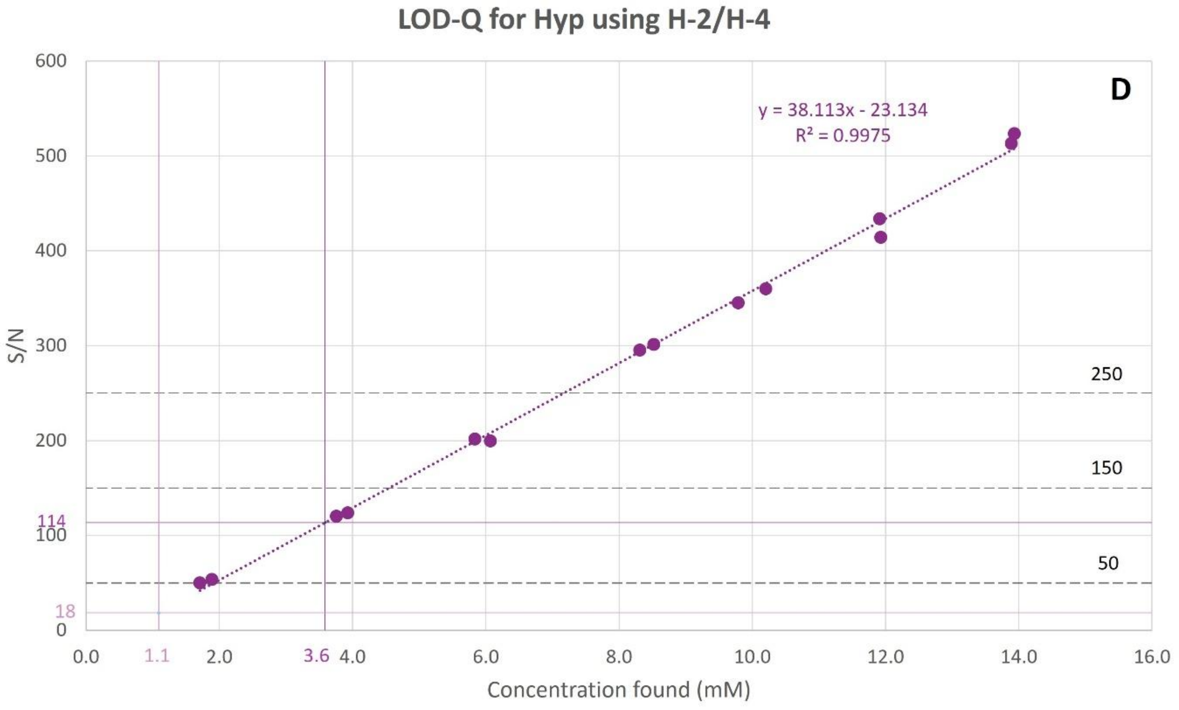
LOD-Q for Hyp using H-2/H-4
D
y = 38.113x - 23.134
R² = 0.9975
250
150
50
114
18
1.1
3.6
S/N
Concentration found (mM)
600
500
400
300
200
100
0
0.0
2.0
4.0
6.0
8.0
10.0
12.0
14.0
16.0

Figure S-6: Stability of the sample TC_E tested over 0.5, 2, 3, 5.5, 10.5, 20, 40, and 72 h, as well at 1, 2, 3, and 9 weeks. All the peak patterns considered in the study were evaluated: Hyp H-2/H-4 and H(*pro-S*)-3, Gly and DSS. The quantification was performed with ERETIC 2, int-qHNMR based method. Average and 1-2-3σ are represented by the dashed lines which are yellow, orange, red and brown respectively.

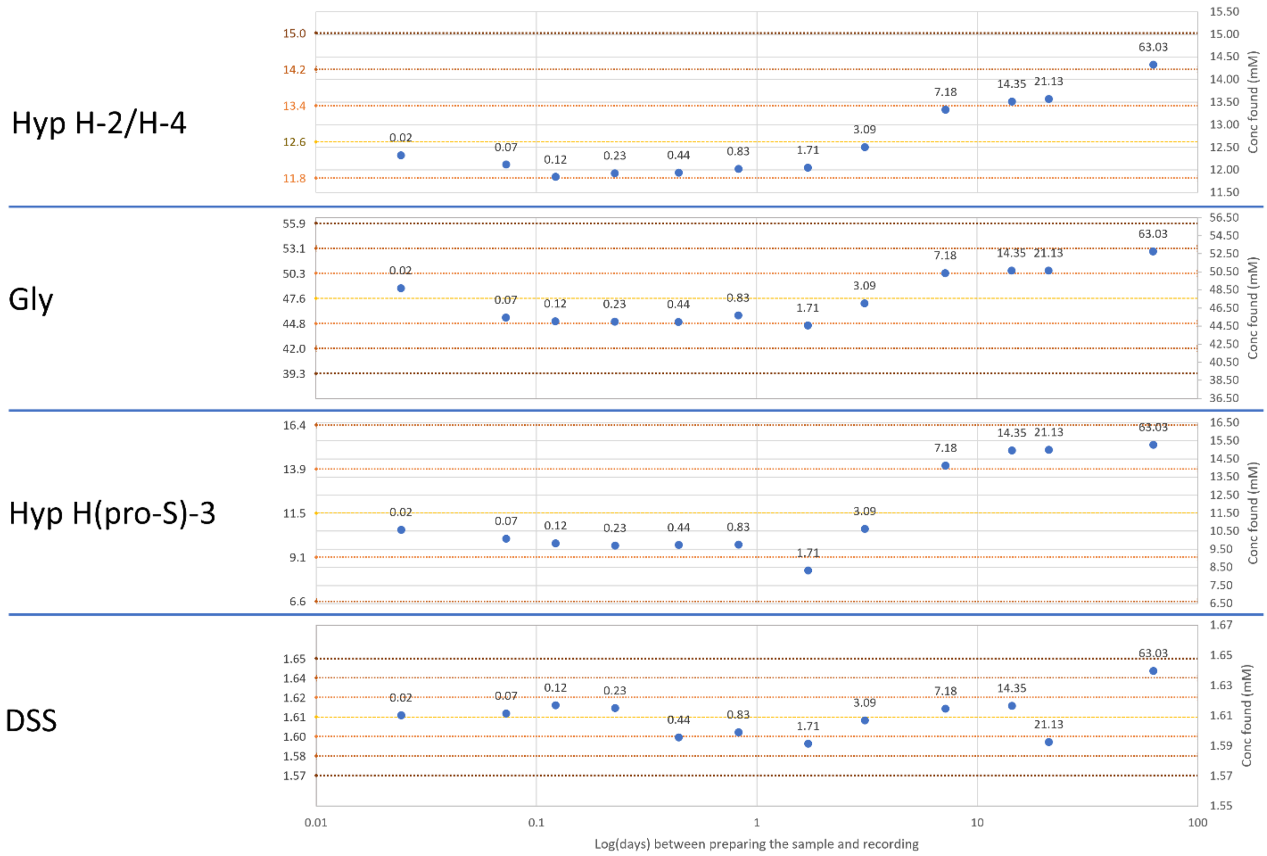

Figure S-7: Stability of the sample TC_E tested over 0.5, 2, 3, 5.5, 10.5, 20, 40, and 72 h, as well at 1, 2, 3, and 9 weeks. All the analytes considered in the study were evaluated: Hyp and Gly, while DSS was used as IC for the ECIC calculations. The quantification was performed with the populations calculated by CT and ECIC, QM-qHNMR based method. Average and 1-2-3σ are represented by the dashed lines which are yellow, orange, red and brown respectively. The red circle highlights the effect of the shimming artifacts in the 40 h spectrum.

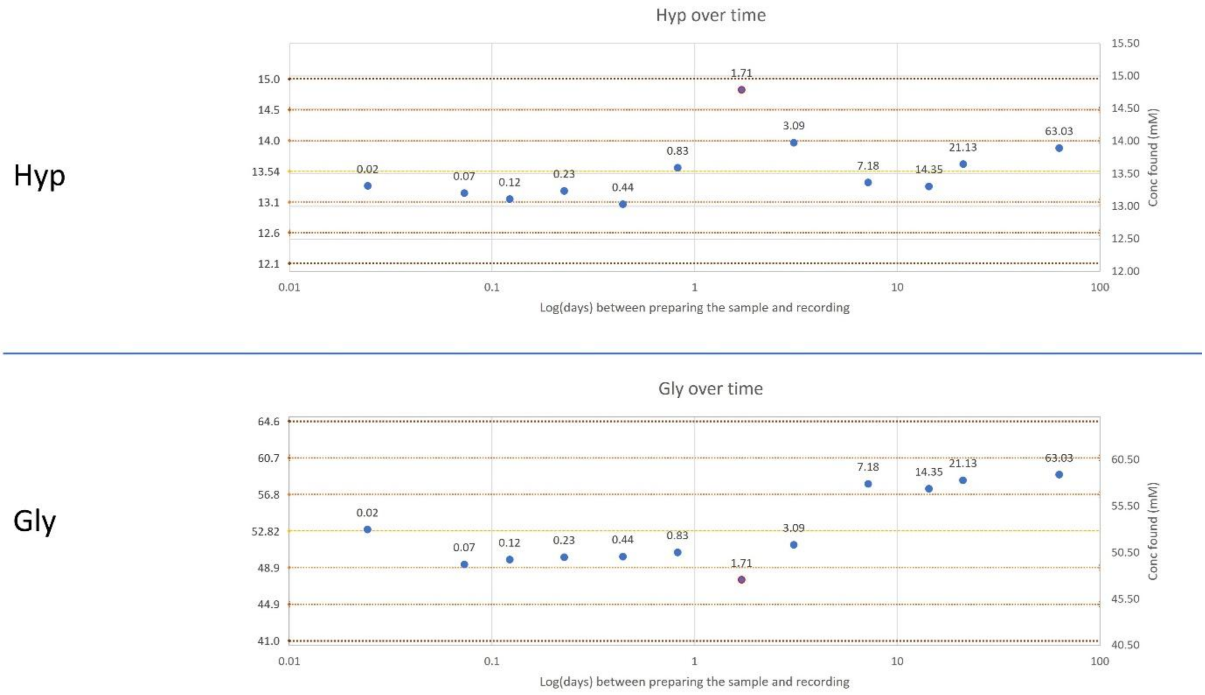

Figure S-8: Shimming quality of the spectra from the stability test. TC_A sample analyzed after 0.5, 2, 3, 5.5, 10.5, 20, 40, and 72 h from its preparation (Figure A, bigger distortion), and 1, 2, 3, and 9 weeks from its preparation (Figure B, smaller distortion). The spectra are zoomed in the region -0.4-0.4 ppm, where the DSS resonance pattern is located, centered at 0.000 ppm.

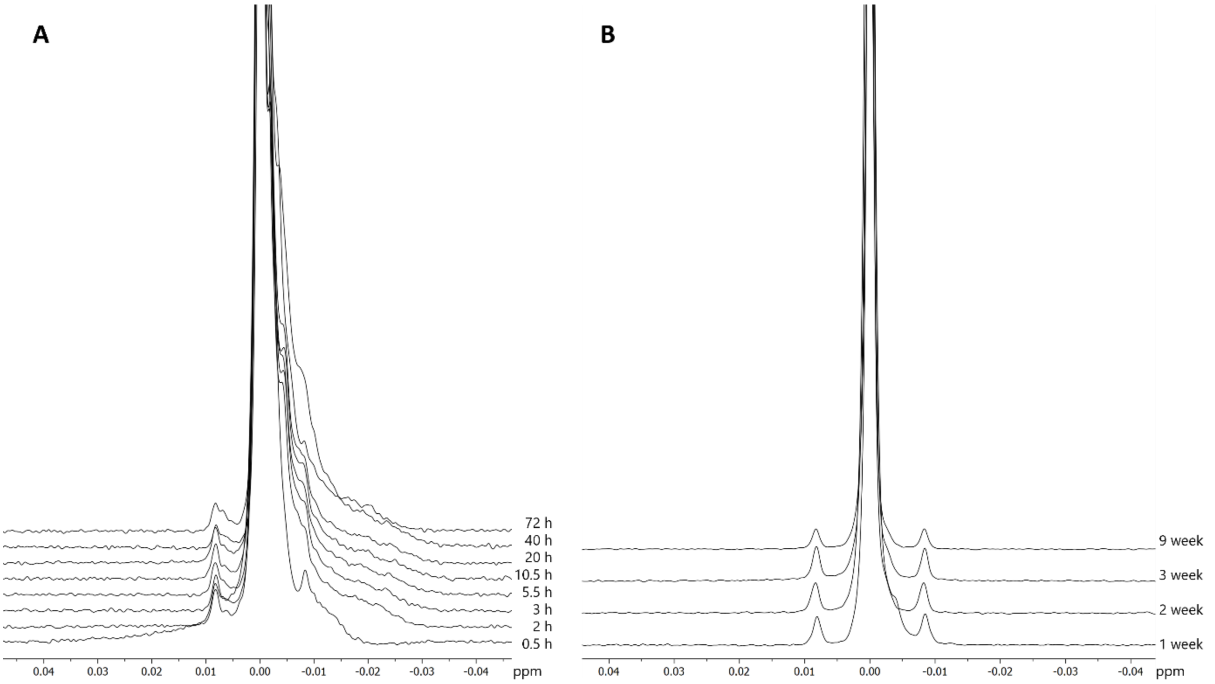

Figure S-9: Solvent peak (HDO) chemical shift (ppm) in relation to the time between the preparation of the sample and the analysis (stability test). In Figure A the time is expressed as days, in Figure B it is expressed as logarithm of the days.

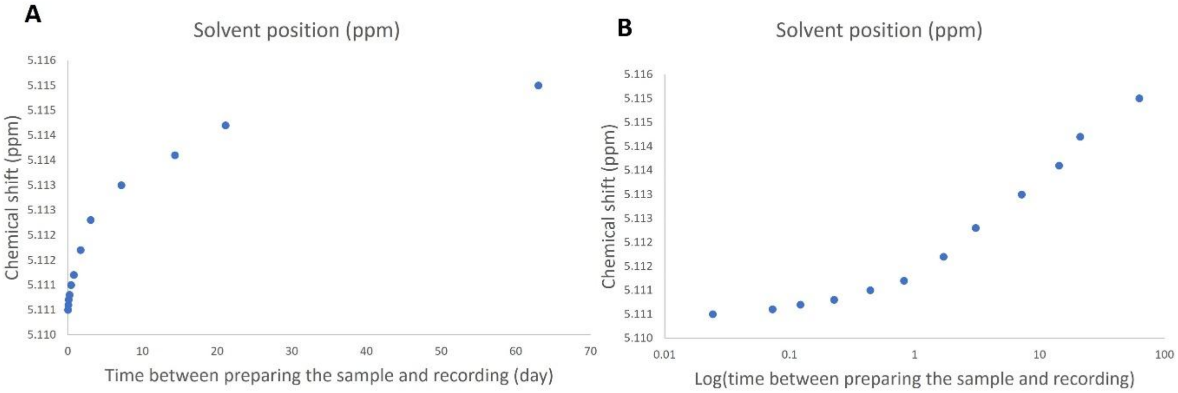

Bibliography

(1) Kristoffersen, K. A.; Afseth, N. K.; Böcker, U.; Dankel, K. R.; Rønningen, M. A.; Lislelid, A.; Ofstad, R.; Lindberg, D.; Wubshet, S. G. Post-enzymatic hydrolysis heat treatment as an essential unit operation for collagen solubilization from poultry by-products. *Food Chemistry* **2022**, *382*, 132201. DOI: 10.1016/j.foodchem.2022.132201.

(2) Lindberg, D.; Kristoffersen, K. A.; De Vogel-Van Den Bosch, H.; Wubshet, S. G.; Böcker, U.; Rieder, A.; Fricke, E.; Afseth, N. K. Effects of poultry raw material variation and choice of protease on protein hydrolysate quality. *Process Biochemistry* **2021**, *110*, 85-93. DOI: 10.1016/j.procbio.2021.07.014.

(3) Pauli, G. F.; Chen, S.-N.; Simmler, C.; Lankin, D. C.; Gödecke, T.; Jaki, B. U.; Friesen, J. B.; McAlpine, J. B.; Napolitano, J. G. Importance of Purity Evaluation and the Potential of Quantitative 1H NMR as a Purity Assay. *Journal of Medicinal Chemistry* **2014**, *57* (22), 9220-9231. DOI: 10.1021/jm500734a.
